# Microbial genetic potential differs among cryospheric habitats of the Damma glacier

**DOI:** 10.1099/mgen.0.001301

**Published:** 2024-10-01

**Authors:** Maomao Feng, Serina Robinson, Weihong Qi, Arwyn Edwards, Beat Stierli, Marcel van der Heijden, Beat Frey, Gilda Varliero

**Affiliations:** 1 Rhizosphere Processes Group, Swiss Federal Institute for Forest, Snow and Landscape Research (WSL), Birmensdorf, Switzerland; 2 Department of Plant and Microbial Biology, University of Zurich, Zurich, Switzerland; 3 Department of Environmental Microbiology, Swiss Federal Research Institute of Aquatic Science and Technology (EAWAG), Dübendorf, Switzerland; 4 Functional Genomics Center Zurich, ETH Zurich and University of Zurich, Zurich, Switzerland; 5 Swiss Institute of Bioinformatics (SIB), Geneva, Switzerland; 6 Department of Life Sciences (DLS), Aberystwyth University, Wales, UK; 7 Plant–Soil Interactions, Agroscope, Zurich, Switzerland

**Keywords:** C-cycling, cryospheric habitats, Damma glacier, functional capability, metagenomic sequencing, N-cycling

## Abstract

Climate warming has led to glacier retreat worldwide. Studies on the taxonomy and functions of glacier microbiomes help us better predict their response to glacier melting. Here, we used shotgun metagenomic sequencing to study the microbial functional potential in different cryospheric habitats, i.e. surface snow, supraglacial and subglacial sediments, subglacial ice, proglacial stream water and recently deglaciated soils. The functional gene structure varied greatly among habitats, especially for snow, which differed significantly from all other habitats. Differential abundance analysis revealed that genes related to stress responses (e.g. chaperones) were enriched in ice habitat, supporting the fact that glaciers are a harsh environment for microbes. The microbial metabolic capabilities related to carbon and nitrogen cycling vary among cryospheric habitats. Genes related to auxiliary activities were overrepresented in the subglacial sediment, suggesting a higher genetic potential for the degradation of recalcitrant carbon (e.g., lignin). As for nitrogen cycling, genes related to nitrogen fixation were more abundant in barren proglacial soils, possibly due to the presence of Cyanobacteriota in this habitat. Our results deepen our understanding of microbial processes in glacial ecosystems, which are vulnerable to ongoing global warming, and they have implications for downstream ecosystems.

**Impact Statement**

Understanding the functional genetic potential of the glacial microbiome is essential for predicting how microbial communities will respond to the melting of glacial ecosystems. Here, we applied shotgun DNA metagenomics to reveal the functional gene diversity and metabolic capabilities of the glacial microbiomes in the Damma glacier, a well-studied glacier in the Swiss Alps. We focused on how the microbial genetic potential varied among different glacial habitats, such as supraglacial and subglacial sediments, basal ice, snow, proglacial stream and (recently deglaciated) barren soil. Collectively, our findings highlight the variation in functional genetic potential among these different glacial habitats. These results represent a further step towards a more comprehensive understanding of the distribution of microbial functional genes in glacial habitats, providing valuable information for targeted biodiversity monitoring and better ecological preservation of these environments.

## Data Availability Statement

All sequence data have been deposited in the National Center for Biotechnology Information NCBI Sequence Read Archive under BioProject PRJNA797782 (https://www.ncbi.nlm.nih.gov/bioproject/ PRJNA797782).

## Introduction

Glacier retreat is a highly visible and widely recognized consequence of global warming, leading to pronounced changes in the diversity and functioning of glacier microbiomes [1,2]. Biologically, the glacial biome is exclusively microbially driven [3], as these organisms are the sole primary producers, in contrast to other terrestrial and freshwater biomes (e.g., forests and rivers). Microorganisms have several strategies to cope with glacial environment characterized by low temperatures, high UV radiation and low nutrient and carbon (C) availability. Whereas adaptations to low temperatures involve altering the membrane lipid compositions and using solutes that are compatible with icy environments to maintain cell turgor and enzymatic functions, adaptations to high UV radiation involve the use of specialized pigments [4,5]. Despite the extreme environmental conditions, the number of microbial cells present in glacial ice and transported to proglacial systems can be considerable; e.g., approximately 1.02×10^21^ cells were calculated to have been transported from the Greenland ice sheet to the downstream fjord in 2012, equivalent to 30.95 mg of carbon [3,6].

Snow is deposited on glacier surfaces through deposition, thereby transferring carbon, nutrients and microorganisms into glacier systems. The melting of the snowpack and glacial ice introduces large amounts of water into the proglacial system during the melt season (i.e. temperate glaciers [7]). During the glacial melt season, temperate glaciers are therefore hydrologically active and the supraglacial habitats are rich in photosynthetically active algae and cyanobacteria, which release C and other nutrients into the glacial system [8,10] and favour the metabolism of heterotrophic microorganisms [11,14]. In subglacial habitats, microbial chemoautotrophy is driven by reduced nitrogen (N), sulphur and iron, and their oxidation is responsible for the dark fixation of inorganic C [4,15, 16]. In addition, microbial chemolithotrophy mediates oxidative weathering of the bedrock in subglacial habitats [17], making chemical energy and limited nutrients available to subglacial microbes [16,18, 19]. Even if spatially distant, the different habitats in glacial ecosystems are interconnected by flowing glacial water [7]. For example, soluble organic C and other nutrients present in the supraglacial meltwater can reach the subglacial habitat through glacial crevasses, moulins and englacial channels [20,21] and then flow to downstream ecosystems, such as glacial forefields and coastal waters [4]. In glacial forefields, the newly exposed barren soils can accumulate nutrients through atmospheric deposition and rock weathering, but the microorganisms in these soils mainly originate from glacial habitats [22].

Glacial microbiomes can further play key roles in element cycling and nutrient accumulation, but these functions have rarely been explored in detail. For example, carbon that enters the glacial system through photoautotrophic and chemoautotrophic microbial activity is the basis of complex food webs in glacial habitats [4]. In addition, C-degrading microbes can result in methane and carbon dioxide accumulation in the subglacial environment, which may be released into the environment after glacial retreat, accelerating global warming [3,23, 24]. Therefore, understanding the taxonomy and functions of glacier microbiomes can help us to better understand the influence of global warming on glacial systems.

The taxonomic composition of microbiomes in glacial and proglacial habitats has been widely reported [22,25,28]. Furthermore, shotgun metagenomic sequencing has been conducted to analyse the microbial functions in subglacial habitats in Alaska [29], glacial cryoconite in the Austrian Alps [30], Himalayan glacial lake sediments [31], the basal ice habitat in an Antarctic glacier [32] and Matanuska Glacier [29], in glaciers spanning from Polar to high-mountain Asian regions [33] and glacier forefield soils of the European Alps [34]. For example, the first metagenomic assembly and bacterial draft genomes were conducted by Kayani *et al*. [29], and the key metabolic pathways such as sulphur oxidation and nitrification were identified. However, most of these studies have focused on one specific cryospheric habitat. How microbial functionality varies among glacial habitats remains largely unknown for temperate glaciers, especially regarding functions related to C degradation, N cycling and chemolithotrophy, which are essential for glacial ecosystems under increasing global warming.

The Damma glacier, located in the Central Alps in Switzerland, has been retreating at an annual rate of ~15 m in the last 15 years [35]. Results from a previous study using amplicon sequencing of bacterial and fungal communities including samples collected from the supraglacial habitat (sediment), subglacial habitat (sediment and ice), proglacial habitat (recently deglaciated soil and proglacial stream water) and surface snow in the Damma glacier environment indicated that bacteria colonizing the recently deglaciated barren soil were mainly derived from the supraglacial, subglacial, proglacial stream and ice habitats, while fungi were more specific to the different habitats [22]. In the present study, we used the same samples to examine how microbial genetic potential and metabolic capabilities differ between the glacial (supraglacial sediment, subglacial sediment and subglacial ice), proglacial (recently deglaciated soil and proglacial stream water) and snow habitats using shotgun metagenomics. To our knowledge, this is the first study using shotgun metagenomic sequencing to compare the microbial functionality between glacial, proglacial and snow habitats. We hypothesized the following:

1. Functional gene diversity and gene structure vary across the glacial, proglacial and snow habitats because of their different environmental characteristics.
2. Genes related to stress responses are more abundant in the glacial ice compared with other habitats because of constant sub-zero temperatures and low nutrient and water availability in ice [4].
3. Genes related to labile C degradation are more abundant in the barren soil and supraglacial and subglacial sediments, as higher concentrations of dissolved organic C have been reported in these habitats [22].
4. Genes related to nitrate reduction (e.g. denitrification) are enriched in the subglacial habitat because its anaerobic conditions favour this process [36].
5. Genes related to rock weathering are present in glacial and proglacial habitats where there is more sediment/soil than ice such as subglacial habitats [19,37].

## Experimental procedures

### Site description, sampling procedure and chemical analyses

The Damma glacier catchment is a well-studied and well-monitored area located in the Swiss Central Alps [38,39]. The climate in the catchment area is characterized by annual precipitation of 2200 mm and significant seasonal temperature fluctuations, ranging from −8 to 4 °C, with an annual average temperature of 2 °C [40,41]. During the summer of 2014, six different habitats were sampled in the Damma glacier catchment. Except for the snow samples, which were taken on 23 June, all other samples were collected on 10 July. Sample collection was described extensively by Rime *et al*. [22], but we give a brief overview of the collection of the samples we analysed in this study. Samples were collected in triplicate from six habitat typologies (Fig. S1, available in the online Supplementary Material):

- Surface snow (‘Snow’): samples of approximately 2 l of surface snow (0–2 cm depth) were collected in triplicate during the melting phase with ethanol-cleaned laboratory spoons in autoclaved polyethylene bags.
- Recently deglaciated soil (‘Soil’): after snow melt, around 100 g of barren soil (0–2 cm depth) was sampled at the same locations, which were marked with sticks during the snow sampling.
- Proglacial stream water (‘Stream’): samples of 2 l of stream water were collected from the water stream at the glacier snout.
- Subglacial ice (‘Ice’): we accessed basal glacier ice by entering a naturally formed cave created by glacier melt. Snow blocked the cave’s entrance until late June before snow melt, ensuring that the ice remained uncontaminated by the atmosphere. Samples of 2 l of ice were collected from the cave at the glacial snout aseptically with an ice pick in autoclaved PE bags.
- Subglacial sediment (‘Sub’): samples of 100 g of subglacial sediment released with ice melt were collected from the cave located at the glacier snout.
- Supraglacial sediment (‘Supra’): samples of 200 g of mineral debris were collected in triplicate from the glacial surface.

All samples were collected with sterile equipment, kept cold during transportation and stored and/or melted at 4 °C overnight. Soil and sediment samples were then stored at −20 °C, whereas ice, snow and glacier stream water samples were filtered through 0.22 µm pore-size water filters (MoBio Laboratories Inc., Carlsbad, CA, USA) for DNA extraction and 0.44 µm pore-size water filters (MoBio) for chemical analyses. The filters (for DNA extraction) and filtrates (for chemical analyses) were then stored at −20 °C until further processing. Detailed information on the geochemical analyses can be found in the study by Rime *et al*. [22].

### DNA extraction and whole shotgun metagenomic sequencing

DNA extraction is detailed by Rime *et al*. [22]. We summarize the protocols here. The total DNA was extracted from soil and sediment samples (Soil, Sub and Supra) using the UltraClean Soil DNA Isolation Kit (MoBio Laboratories Inc.), while DNA was extracted from the filters (Snow, Ice and Stream) using the RapidWater DNA Isolation Kit (MoBio) according to the manufacturer’s protocol. DNA was quantified with PicoGreen (Invitrogen, Carlsbad, CA, USA) and stored at −20 °C. As the sample ‘Ice’ yielded small amounts of DNA, four extractions per ice sample were pooled to form one independent sample. Shotgun library preparation was carried out with a Nextera XT Library Preparation Kit (Illumina, San Diego, CA, USA). Sequencing reactions were carried out on the Illumina HiSeq 2500 platform (2×100 cycles) at the IBERS Aberystwyth Translational Genomics Facility. Three metagenomic samples were obtained per habitat. However, one of the samples from ‘Ice’ (Ice_3) was excluded from further analysis due to the small number of yielded sequences. The raw sequences were deposited in the NCBI Sequence Read Archive under accession number PRJNA797782. Detailed information on the 16S rRNA and internal transcribed spacer (ITS) gene copies can be found in Rime *et al*. [22].

### Metagenome assembly and functional annotation

A customized pipeline was applied to process raw reads, assemble them into contigs and annotate contigs for functionality and taxonomy [42]. In short, the quality of raw reads was checked using FastQC v0.11.8 [43], followed by quality filtering, read trimming and removal of Illumina adapters using Trimmomatic v0.36 (*Q*=20, MINLEN=40) [44], which resulted in pre-processed reads. The pre-processed reads were then assembled into contigs (>200 bp) with MEGAHIT v1.2.9, by iteratively building de Bruijn graphs using k-mers of increasing size (-k-min 27, -k-step 10 [45]). MetaGeneMark v3.38 was used to predict protein-coding genes in the contigs [46]. The predicted genes were annotated to the eggNOG [47], CAZy [48] and NCyc [49] databases. eggNOG, which classifies genes into orthologous groups (OGs) of proteins and assigns OGs to general functional categories, was used to evaluate the microbial genetic potential for general metabolic and cellular functions. The annotation to the eggNOG v4.5 database was carried out with eggNOG-mapper v1.0.3 operated with the DIAMOND search mode against all protein sequences [50]. C- and N-cycling genes were annotated with the CAZy and NCyc databases to reveal the microbial genetic potential related to the C cycle and N cycle, respectively. The annotation of the predicted genes to the CAZy (downloaded on 20 July 2017) and NCyc (curated sequences clustered at 100% sequence identity) databases was done with SWORD v1.0.3 (-v 10–6) [51]. In addition to the categorization by enzyme class implemented in CAZy, a manual categorization of CAZy genes into different C substrates was performed as previously outlined [42,52].

We identified weathering genes associated with siderophore, oxalate and cyanide synthesis in our study. The method has also been used by Varliero *et al*. [53]. The *obcA* genes are involved in the first step of oxalate biosynthesis [54]. The production of organic acids (e.g. oxalate) and hydrogen cyanide (HCN) by weathering-enhancing organisms has been observed to mobilize nutrients such as iron, sulphur and phosphorus [55], and an increase in siderophore production can help import iron into the cell [56,58].

### Abundance quantification of protein-coding genes

BWA aligner v0.7.15 (bwa-mem [59]) was used to map pre-processed read pairs to assembled contigs. Gene abundances were obtained by counting the reads that mapped to the predicted protein-coding genes using the ‘featureCounts’ function from the Subread package v2.0.1 (-minOverlap 10, *Q*=10, -primary [60]).

### Taxonomic annotation

The predicted protein-coding genes annotated to the functional databases (e.g. eggNOG) were assigned taxonomically using Kaiju v1.7.4 [61] with the NCBI blast nr+euk database (created on 24 May 2023) and default settings. The helper programme kaiju-addTaxonNames was utilized to convert NCBI taxon IDs into taxonomy. Additionally, the CheckM v1.1.2 [62] function ‘-ssu_finder’ was used to identify 16S and 18S ribosomal DNA (rDNA) sequences from the contigs. Small subunit ribosomal RNA (SSU rRNA) sequences were assigned to the silva taxonomy database (release 138 [63]) using sina v1.2.12 [64]. To estimate the abundances of the 16S and 18S rRNA genes, the corresponding read counts per contig were normalized to the contig length in kbp.

### Statistical analyses

Alpha-diversity indices of functional and taxonomic genes were analysed based on sequences rarefied to even depth corresponding to the total number of reads associated with the smallest sample (*n*=3 337 869), using the phyloseq package [65] in R v4.3.1 (R Core Team 2023) [66]. Differences between different habitats in soil properties, relative abundances of different phyla, relative abundance of weathering genes and alpha-diversity indices of different functional and taxonomic genes were assessed by one-way ANOVA followed by a least significant difference (LSD) test. Multiple comparison tests were conducted using the ‘LSD.test’ function from the R package agricolae [67]. To investigate the differences in functional and ribosomal gene structures, principal coordinate analysis (PCoA) based on Bray–Curtis dissimilarity matrices of DESeq2 [68,69] normalized counts was applied for the CAZy, NCyc and eggNOG datasets and of relative abundances for ribosomal genes. PCoA was implemented using the ‘pcoa’ function in the R package ape [70]. To assess the relationship between soil properties and 16S rRNA and ITS gene copy numbers with functional and ribosomal community structures, these data were regressed against the PCoA ordination scores using the ‘envfit’ function in the R package vegan [71]. The bacteria-to-fungi ratio (16S/ITS) was calculated using the ratio of 16S rRNA to ITS copy numbers and provides an indication of organic matter decomposition and nutrient mineralization rates [72]. This is due to the differing rates of decomposition and mineralization in bacteria-dominated versus fungi-dominated soils, with higher rates typically observed in the former [72]. Permutational multivariate ANOVA was performed in PRIMER v7 [73] to assess the significance of differences in community structure among the habitats. Differences in multivariate dispersion among functional and taxonomic communities were analysed using the PERMADISP function in PRIMER v7. To assess the changes in functional genes (annotated using eggNOG, CAZy and NCyc) across the different habitats, the log_2_ fold changes in functional genes between each two habitats were calculated using the R package DESeq2 [68,69]. Data manipulation was done using the R packages dplyr [74], plyr [75] and reshape2 [76]. All plots were generated with the R package ggplot2 [77].

## Results

### Changes in environmental and microbial characteristics among habitats

All soil physicochemical variables differed among the habitats, except for nitrite and phosphate concentrations (Table S1 and Fig. S2). Soil pH ranged from 5.2 to 6.7, with the highest pH occurring in ‘Supra’ and the lowest in ‘Soil’ and ‘Sub’ (Table S1). Dissolved organic carbon (DOC) concentrations ranged from 0.003 µg g^−1^ in ‘Stream’ to 17.1 µg g^−1^ in ‘Soil’ samples. Dissolved organic nitrogen (DON) concentrations ranged from 0.08 µg g^−1^ in ‘Ice’ to 1.05 µg g^−1^ in ‘Soil’. Regarding biotic (microbial) properties, 16S rRNA gene copy numbers were largest in ‘Soil’ and smallest in ‘Ice’. The highest 16S rRNA to ITS ratio occurred in ‘Sub’, while there was no significant difference among the other habitats.

### Assembly statistics

After quality control, 324 761 303 high-quality reads (7 355 598 to 34 517 135 reads per sample) were obtained (Tables S2 and S3). The MEGAHIT assembly of reads into contigs produced a total of 2 443 106 contigs of 802 bp on average, ranging from 200 to 419 451 bp with an N50 value of 1000 and a GC content of 46% (Table S4). In total, 3 337 869 predicted genes were found among the contigs, 1 595 443 of which could be annotated with the general eggNOG database, 33 369 with CAZy and 5251 with NCyc, corresponding to 47.8, 1.0 and 0.16% of the reads mapped to predicted genes, respectively (Table S4).

### Taxonomic composition of the metagenomes

**Fig. 1.**
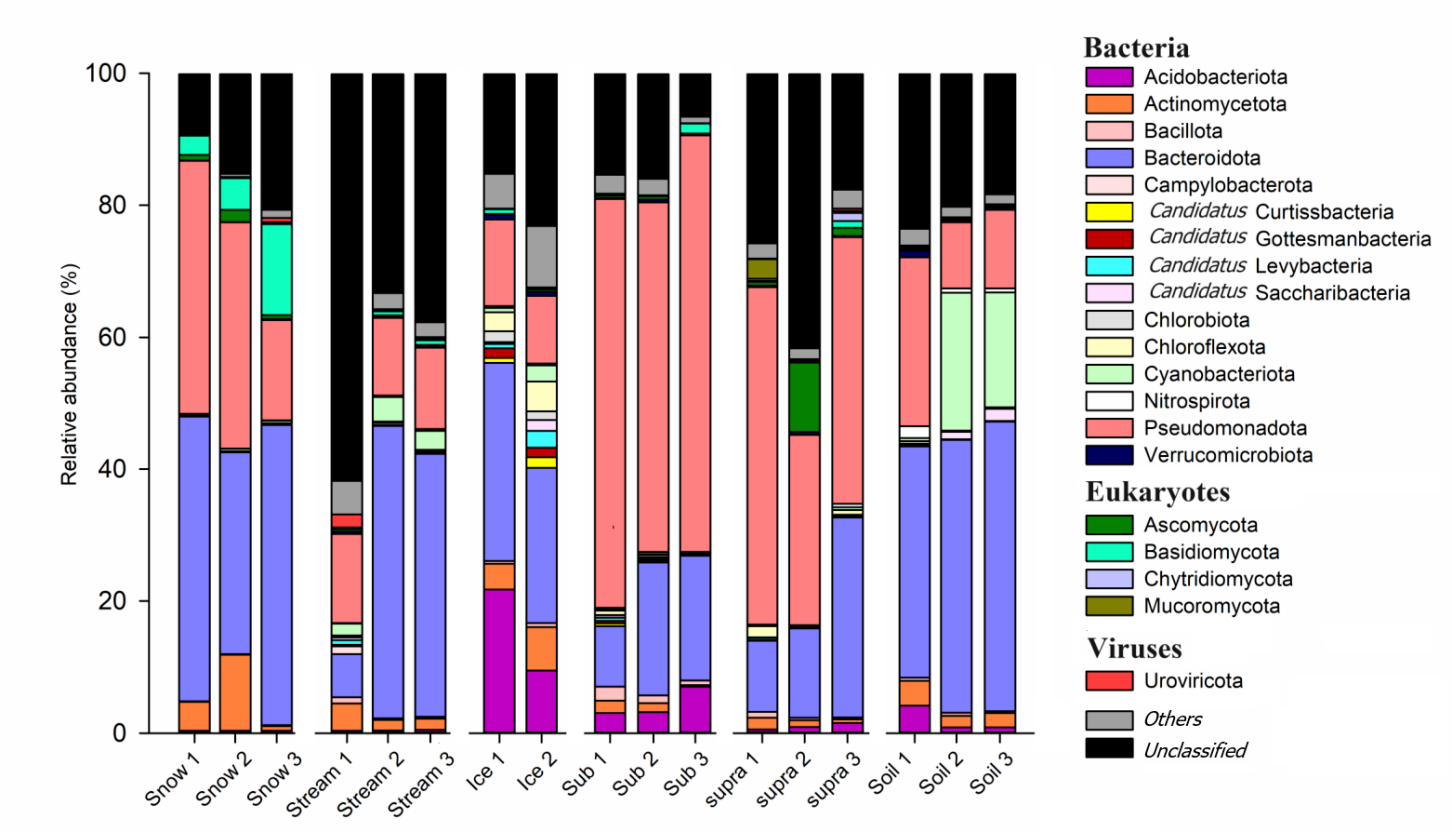
Taxonomic composition of the microbiomes in six Damma glacial habitats based on predicted genes. Relative abundance is the mean of three replicates (only two replicates for ice samples). Only phyla with a relative abundance >1% in at least one habitat are shown. Snow, surface snow; Ice, subglacial ice; Stream, proglacial stream water; Sub, subglacial sediment; Supra, supraglacial sediment; Soil, recently deglaciated soil.

Bacteria were the most abundant organisms in all habitats, with relative abundances higher than 50%, except for one sample in ‘Stream’, where more than 60% of the sequences were unclassified (Figs 1 and S3). The highest percentages of eukaryotes were found in the ‘Snow’ and ‘Supra’ habitats, with 8.4 and 5.7%, respectively (Table S5). The other habitats were represented by 0.5–0.88% reads assigned to eukaryotes. Among the bacteria, the phylum Pseudomonadota (31% on average) dominated the predicted genes, followed by Bacteroidota (26% on average), Acidobacteriota (4.3% on average), Actinomycetota (3.1% on average) and Cyanobacteriota (1.0% on average) (Table S5). The relative abundance of Pseudomonadota was especially high in ‘Sub’ (59% on average), while the relative abundance of Bacteroidota was highest in ‘Soil’ (40% on average). Cyanobacteriota was most abundant in ‘Soil’ (13% on average) (Table S5). For eukaryotes, a higher abundance of Ascomycota was found in the ‘Supra’ (4.1% on average) and ‘Snow’ (1.1% on average) (Table S5). Detailed descriptions of the taxonomical classification of the different datasets are given in Table S5.

### Shifts in diversity and structure of functional genes among habitats and linkages with environmental variables

There was a significant effect of habitat on the richness of the functional and ribosomal genes annotated using CAZy and NCyc databases (Fig. S4 and Table S6), with the highest values in ‘Soil’ and the lowest in ‘Sub’ for all three databases. Details of the alpha diversity of functional genes annotated with eggNOG, CAZy and NCyc at the category/class/family level can be found in the Supplementary Material (Figs S5–S10).

**Fig. 2.**
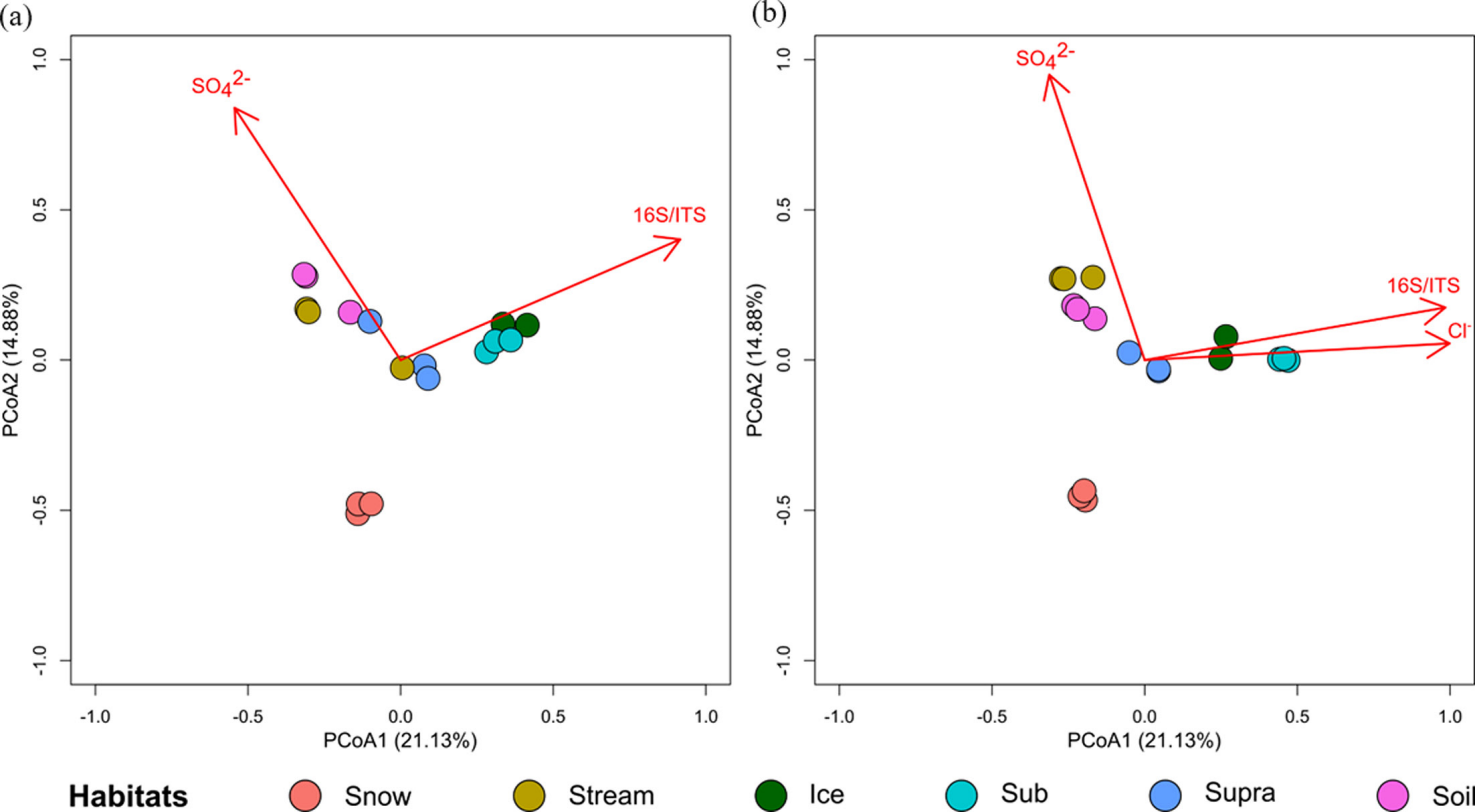
Changes in functional (**a**) and SSU rRNA (**b**) gene structure among six Damma glacial habitats. The percentage of the variation explained by each PCoA axis is given in brackets. Vectors represent a regression of soil physicochemical and biotic parameters against the PCoA ordination scores. All the vectors shown here had significant correlations (*P*<0.05), with microbial functional and ribosomal gene structures. Snow, surface snow; Ice, subglacial ice; Stream, proglacial stream water; Sub, subglacial sediment; Supra, supraglacial sediment; Soil, recently deglaciated soil. SO_4_^2−^, sulphate; Cl^−^, chloride; 16S/ITS, the ratio of copy numbers of bacterial 16S rRNA gene and fungal ITS.

There was also a significant effect of habitat on the structure of functional (eggNOG: *P=*0.0001; CAZy: *P=*0.0001 and NCyc: *P=*0.0001) and SSU rRNA genes (*P=*0.0001; Table 1). Specifically, structures of all functional and ribosomal genes were significantly different for the ‘Snow’ and ‘Ice’, ‘Snow’ and ‘Sub’, ‘Snow’ and ‘Soil’, ‘Ice’ and ‘Soil’ and ‘Sub’ and ‘Soil’ pairwise habitat comparisons (Table 1). In addition, the eggNOG- and NCyc-annotated functional and SSU rRNA community structures differed significantly in the ‘Snow’ and ‘Stream’ habitat comparison (Table 1). The beta dispersion results further suggested that the differences in microbial gene structure between habitats were not caused by the within-group difference (Table S7). Results of the ‘envfit’ analysis suggest that the functional gene structure was significantly (*P*<0.05) correlated with the SO_4_^2−^ concentration and the 16S/ITS ratio (Figs 2 and S11 and Table S8). When looking at the SSU rRNA gene dataset, SO_4_^2−^ concentrations, Cl^−^ concentrations and the 16S/ITS ratio were significantly correlated. ‘Snow’ formed a cluster distinct from the other samples. ‘Ice’ and ‘Sub’ clustered closely together, whereas samples from ‘Supra’, ‘Stream’ and ‘Soil’ formed a separate cluster (Figs 2 and S11).

**Table 1.** Changes in functional (annotated using eggNOG, CAZy and NCyc databases) and SSU rRNA gene structure of microbiomes in the six Damma glacial habitats. Significance was tested by permutational multivariate ANOVA (PERMANOVA). Values are means±sd (*n*=3, except for ‘Ice’ where *n*=2). Significant tests (*P*<0.05) are marked in bold

	eggNOG		CAZy		NCyc		SSU rRNA	
**Main effect**								
	*F*	*P*	*F*	*P*	*F*	*P*	*F*	*P*
Habitat	2.81	**0.0001**	2.63	**0.0001**	3.01	**0.0001**	3.67	**0.0001**
**Habitat**								
Snow vs. Stream	1.90	**0.02**	1.85	0.06	2.03	**0.04**	2.45	**0.03**
Snow vs. Ice	2.64	**0.02**	0.10	**0.04**	0.10	**0.02**	3.00	**0.02**
Snow vs. Sub	2.29	**0.02**	0.10	**0.04**	0.10	**0.02**	2.51	**0.01**
Snow vs. Supra	1.67	0.09	1.62	0.08	1.77	0.06	1.75	0.07
Snow vs. Soil	2.51	**0.01**	2.35	**0.02**	2.42	**0.01**	3.05	**0.01**
Stream vs. Ice	1.60	0.13	0.10	0.13	0.10	0.10	2.05	0.06
Stream vs. Sub	1.76	0.06	0.10	0.09	0.10	0.06	2.02	**0.03**
Stream vs. Supra	1.29	0.21	1.23	0.24	1.23	0.25	1.36	0.18
Stream vs. Soil	1.41	0.16	1.51	0.13	1.46	0.15	2.07	**0.04**
Ice vs. Sub	1.61	0.11	0.19	0.26	0.10	0.16	1.53	0.14
Ice vs. Supra	1.35	0.21	0.20	0.22	0.20	0.18	1.36	0.20
Ice vs. Soil	2.15	**0.04**	2.22	**0.04**	2.20	**0.04**	2.52	**0.02**
Sub vs. Supra	1.30	0.21	0.09	0.20	0.10	0.14	1.36	0.17
Sub vs. Soil	2.12	**0.03**	0.10	**0.04**	0.10	**0.04**	2.42	**0.02**
Supra vs. Soil	1.54	0.14	1.54	0.11	1.58	0.10	1.66	0.09

Icesubglacial iceSnowsurface snowSoilrecently deglaciated soilStreamproglacial stream waterSubsubglacial sedimentSuprasupraglacial sediment

### Shifts in eggNOG genes across different habitats

For pairwise comparisons, we only report the ones that most differed at the individual gene level or functional category level and that have ecological implications. It is the same for the CAZy and NCyc datasets.

**Fig. 3.**
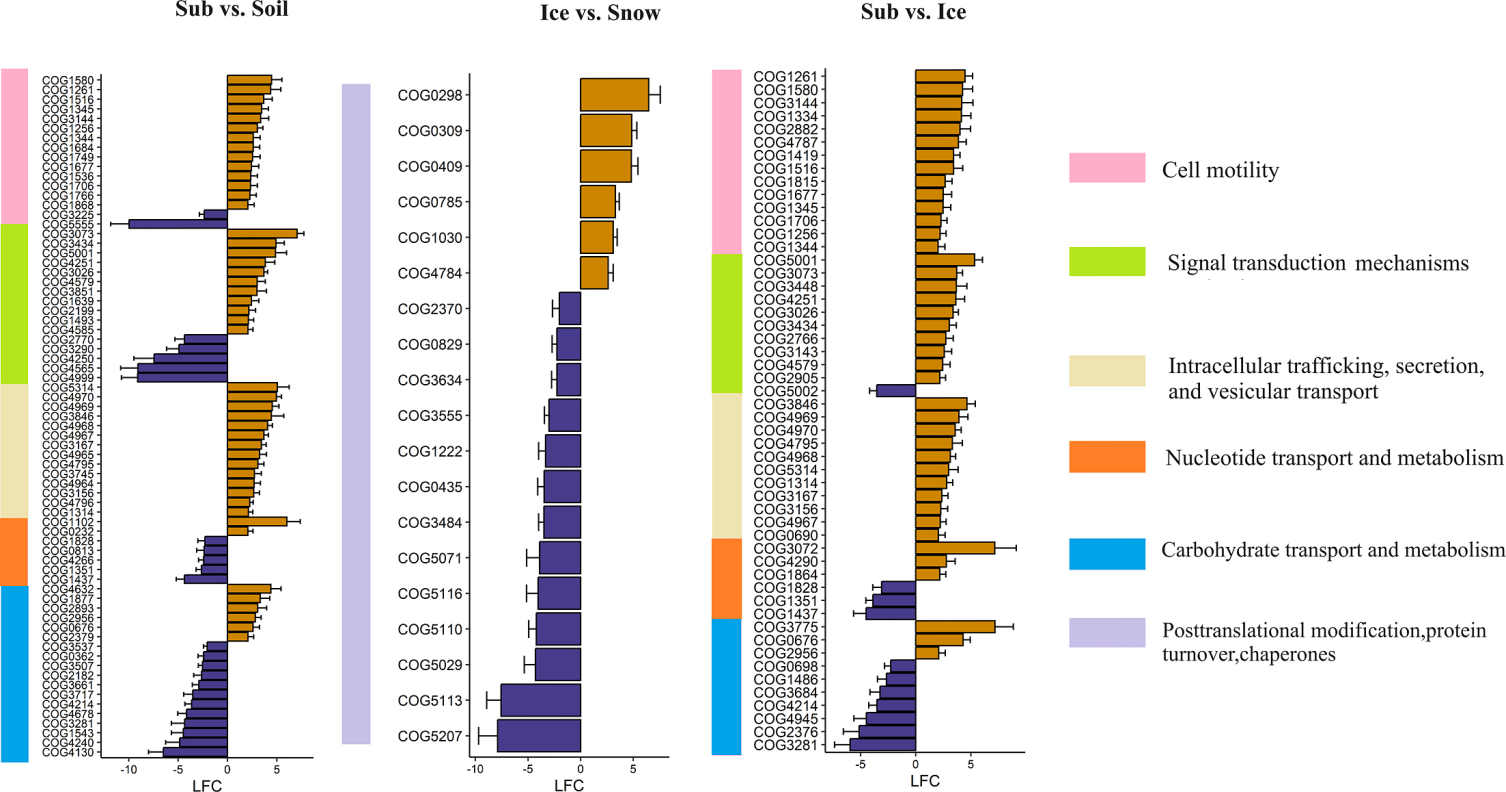
Differentially abundant functional genes between Damma glacier habitats. Bars represent the log_2_ fold change (LFC) in functional genes annotated using the eggNOG database between the Sub and Soil, Ice and Snow and Sub and Ice habitats. Only significantly (*P* < 0.05) differentially abundant genes with |LFC| > 2 are depicted. Only COGs in the category ‘posttranslational modification, protein turnover and chaperones’ are presented for ‘Ice vs. Snow’. Orange indicates overrepresented COGs (LFC > 0), and violet indicates underrepresented COGs (LFC < 0). The log_2_ fold change (LFC) value ‘Sub and Soil’ is the log_2_ of gene abundance in Sub/gene abundance in Soil. The same holds for the ‘Ice and Snow’ and ‘Sub and Ice’ habitats. Snow, surface snow; Ice, subglacial ice; Snow, surface snow; Soil, recently deglaciated soil.

The comparison between ‘Sub’ and ‘Soil’ and ‘Sub’ and ‘Ice’ showed that several genes were enriched in ‘Sub’, such as genes related to ‘cell motility’ (e.g. COG1580: flagellar basal body-associated protein; COG1677: flagellar hook-basal body complex protein FliE and COG3144: flagellar hook-length control protein), ‘intracellular trafficking, secretion and vesicular transport’ (e.g. COG4967: type IV fimbrial biogenesis; COG4968: type IV fimbrial biogenesis transmembrane protein and COG4970: type II transport protein GspH) and ‘signal transduction mechanisms’ (e.g. COG3434: signal transduction protein; COG4251: transduction histidine kinase; and COG5001: diguanylate cyclase phosphodiesterase) (Figs 3 and S12 and Table S16).

Comparisons between ‘Ice’ and ‘Snow’ showed that ‘Ice’ was enriched with genes within the category ‘posttranslational modification, protein turnover and chaperones’, such as COG0298 (hydrogenase expression formation protein), COG0785 (cytochrome C biogenesis) and COG1030 (membrane-bound serine) (Fig. 3 and Table S16).

For detailed information on the composition of functional genes and functional genes/categories that differed significantly in abundance between each two habitats, please refer to Tables S9 and S10 and Supplementary Results.

### Changes in C-degrading genes across different habitats

**Fig. 4.**
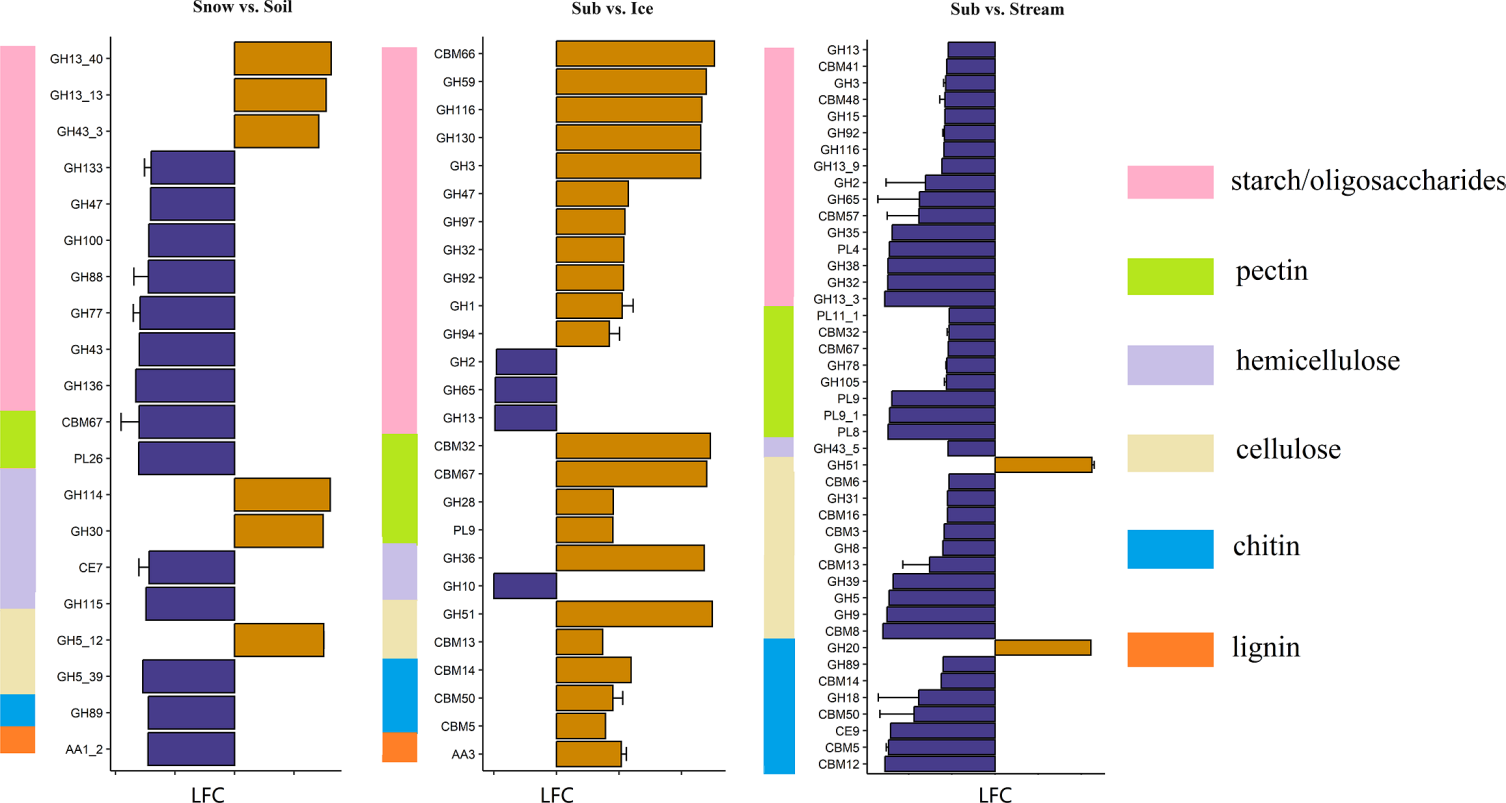
Differentially abundant C-degrading genes between Damma glacier habitats. Bars represent the log_2_ fold changes (LFCs) in C-degrading genes annotated using the CAZy database between the Snow and Soil, Sub and Ice and Sub and Stream habitats. Only significantly (*P* < 0.05) differentially abundant genes with |LFC| > 7 are depicted. Orange indicates overrepresented genes (LFC > 0), and violet indicates underrepresented genes (LFC < 0). The log_2_ fold change (LFC) value ‘Snow and Soil’ is the log_2_ of gene abundance in Snow/gene abundance in Soil. The same holds for the ‘Sub and Ice’ and ‘Sub and Stream’ habitats. For example, GH13_40 was overrepresented in Snow relative to Soil, while CH133 was more abundant in Soil than in Snow. All the genes and their annotations can be found in Table S11. GH, glycoside hydrolase; CBM, carbohydrate-binding module; PL, polysaccharide lyase; CE, carbohydrate esterase. Snow, surface snow; Ice, subglacial ice; Stream, proglacial stream water; Sub, subglacial sediment; Soil, recently deglaciated soil.

Comparisons of the CAZy enzyme classes between ‘Sub’ and ‘Soil’, ‘Sub’ and ‘Ice’ and ‘Sub’ and ‘Stream’ showed that auxiliary activities (AAs) were more abundant in ‘Sub’, such as AA3, which is involved in the degradation of lignin (Figs 4 and S12 and Table 2). Polysaccharide lyases (PLs) were underrepresented in ‘Sub’ (Table S2), such as PL9, which is involved in the degradation of pectin (Fig. 4).

**Table 2.** Summary of the functional genes annotated using CAZy and NCyc databases that were significantly (*P*<0.05) overrepresented/underrepresented in one specific habitat compared to all the other Damma glacier habitats. ‘↑’ indicates genes that are significantly more abundant (LFC > 0, *P*<0.05) in the indicated habitat compared to all other habitats, ‘↓’ indicates genes that were significantly less abundant (LFC < 0, *P*<0.05) in the indicated habitat. We indicate in brackets the target substrates on the CAZy enzymes. For example, the gene ‘AA3’, encoding for the enzyme alcohol oxidase and belonging to the family ‘AA’, is overrepresented in Snow

Habitat	Database	Family	Gene ID	Enzyme/function
Snow	CAZy	AA ↑	AA3	Alcohol oxidase (lignin/cellulose)
		GH ↑	GH3	*β*-Glucosidase (cellooligosaccharides)
			GH23	Peptidoglycan lyase (murein)
			GH2	*β*-Galactosidase (oligosaccharides)
		PL ↓	PL1_2	Pectate lyase (pectin)
	NCyc	ANR ↑	narB	Assimilatory nitrate reductase
		Nitrification ↓	amoB_B	Ammonia monooxygenase subunit B
Stream	CAZy	PL ↑	PL1_2	Pectate lyase (pectin)
		AA ↓	AA3	Alcohol oxidase (lignin/cellulose)
	NCyc	OD&S ↓	glnA	Glutamine synthetase
Ice	CAZy	AA ↓	AA3	Alcohol oxidase (lignin/cellulose)
		GH ↓	GH92	Mannosyl-oligosaccharide (oligosaccharides)
			GH3	*β*-Glucosidase (cellooligosaccharides)
			GH23	Peptidoglycan lyase (murein)
	NCyc	Denitrification and DNR ↑	nirB	Nitrite reductase (NADH) large subunit
			narG	Nitrate reductase
		ANR ↑	NR	Nitrate reductase (NAD(P)H)
			nasA	Assimilatory nitrate reductase catalytic subunit
		Nitrification ↑	hao	Hydroxylamine dehydrogenase
			amoC_B	Ammonia monooxygenase subunit C
Sub	CAZy	AA ↑	AA3	Alcohol oxidase (lignin/cellulose)
		PL ↓	PL8	Exo-*β*-1,4-glucuronan lyase
			PL26	Rhamnogalacturonan exolyase (pectin)
	NCyc	\		
Supra	CAZy	GT ↑	GT4	Sucrose synthase
	NCyc	\		
Soil	CAZy	CE ↑	CE1	Acetyl xylan esterase (hemicellulose)
			CE4	Acetyl xylan esterase (hemicellulose)
		GH ↑	GH2	*β*-Galactosidase (oligosaccharides)
			GH3	*β*-Glucosidase (cellooligosaccharides)
	NCyc	NF ↑	nifH	ATP-binding iron-sulphur protein
			nifD	Nitrogenase molybdenum-iron protein alpha chain
		Nitrification ↑	amoC_B	Ammonia monooxygenase subunit C (bacteria)

ANRassimilatory nitrate reductionCEcarbohydrate esterasesDNRdissimilatory nitrate reductionGHglycoside hydrolasesGTglycosyl transferasesIcesubglacial iceNFnitrogen fixationOD&Sorganic degradation and synthesisPLpolysaccharide lyasesSnowsurface snowSoilrecently deglaciated soilStreamproglacial stream waterSubsubglacial sedimentSuprasupraglacial sediment

For detailed information on C-degrading classes/families that were differentially abundant between each two habitats, please refer to Tables S9 and S11 and Supplementary Results.

### Changes in N-cycling genes across different habitats

**Fig. 5.**
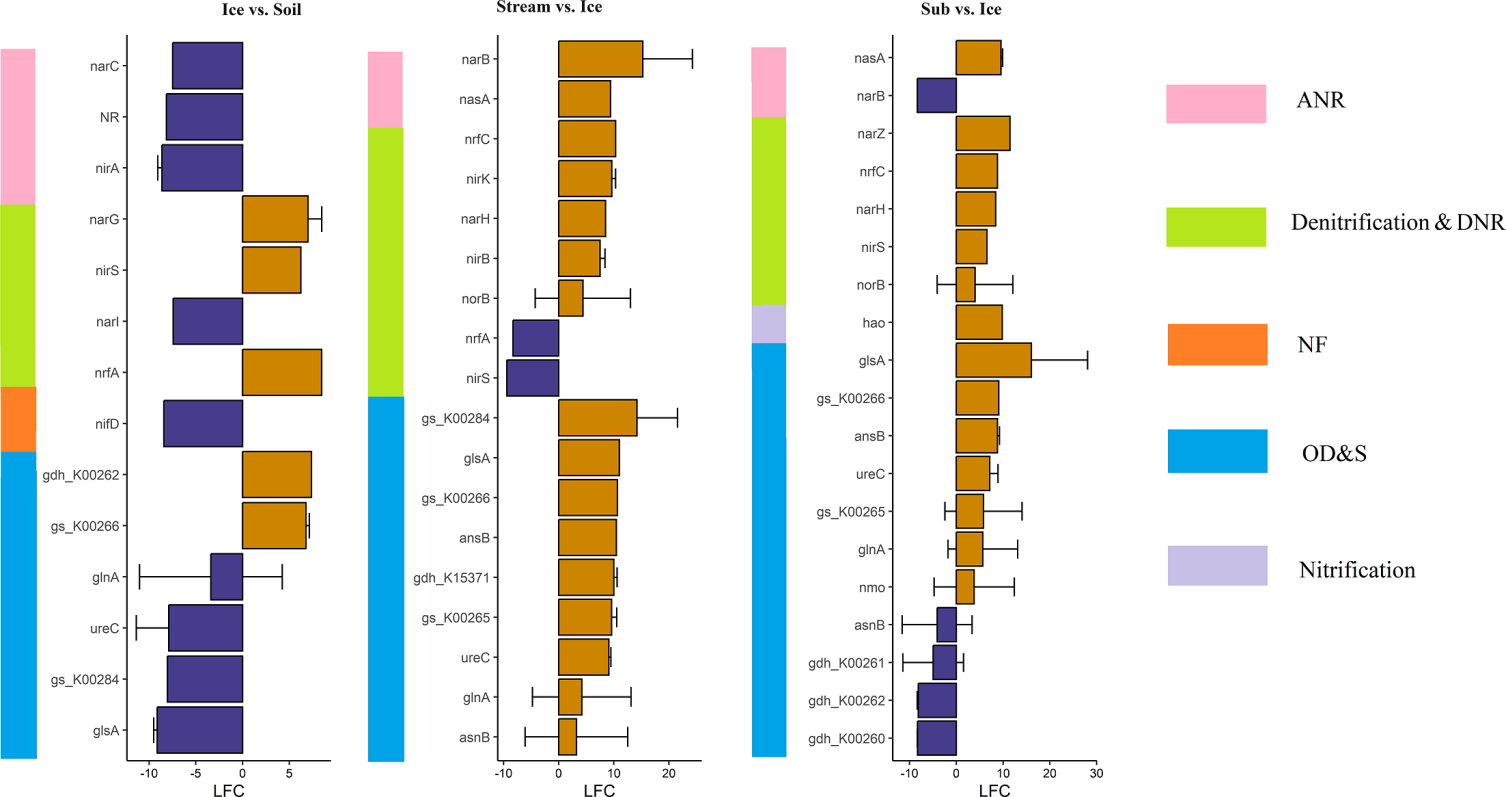
Differentially abundant N-cycling genes between Damma glacial habitats. Bars represent log_2_ fold changes (LFCs) in N-cycling genes annotated using the NCyc database between the Ice and Soil, Stream and Ice and Sub and Ice habitats. Only significantly (*P*<0.05) differentially abundant genes with |LFC| > 3 are depicted. Orange indicates overrepresented genes (LFC > 0) and violet indicates underrepresented genes (LFC < 0). The log_2_ fold change (LFC) value ‘Ice and Soil’ is the log_2_ of gene abundance in Ice/gene abundance in Soil. The same holds for the ‘Stream and Ice’ and ‘Sub and Ice’ habitats. For example, *narC* was overrepresented in Ice relative to Soil (**a**). All the genes and their annotations can be found in Table S12. DNR, dissimilatory nitrate reduction; NF, nitrogen fixation; OD&S, organic degradation and synthesis; Ice, subglacial ice; Stream, proglacial stream water; Sub, subglacial sediment; Soil, recently deglaciated soil.

Comparisons of the NCyc families between ‘Ice’ and ‘Soil’, ‘Stream’ and ‘Ice’ and ‘Sub’ and ‘Ice’ showed that N-cycling genes related to assimilatory nitrate reduction (ANR) were enriched in the ‘Ice’ habitat, such as *narB*, *narC*, *NR* and *nirA* (Figs 5 and S12).

In addition, comparisons of the NCyc families between ‘Ice’ and ‘Soil’ and ‘Supra’ and ‘Soil’ suggested that genes related to nitrogen fixation were enriched in ‘Soil’ habitat, such as *nifD* (Figs 5 and S12 and Table 2).

For detailed information on N-cycling families/genes that were differentially abundant between each two habitats, please refer to Tables S9 and S12 and Supplementary Results.

### Microorganisms residing in snow and subglacial sediments have a high capacity for rock weathering

**Fig. 6.**
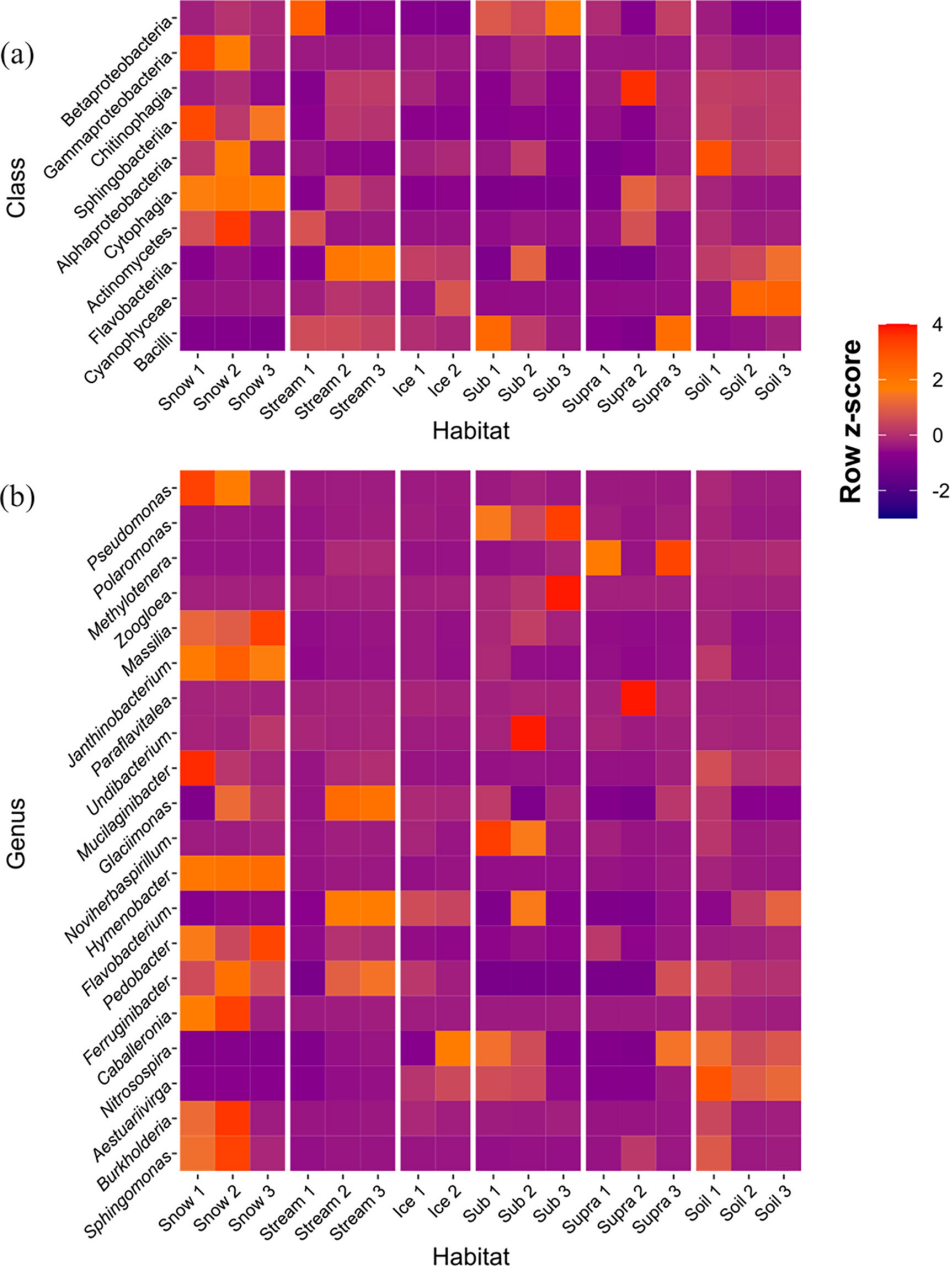
Row z-scores of weathering-associated microorganisms at the class (**a**) and genus (**b**) levels in the six Damma glacial habitats. Classes with relative abundances ranking in the top 10 and genera with relative abundances ranking in the top 20 are shown. The sample IDs are formatted as habitat followed by a replicate number. Snow, surface snow; Ice, subglacial ice; Stream, proglacial stream water; Sub, subglacial sediment; Supra, supraglacial sediment; Soil, recently deglaciated soil.

We identified weathering genes associated with siderophore, oxalate and cyanide synthesis in our glacial samples [53], which have been observed to mobilize nutrients such as iron, sulphur and phosphorus (oxalate and cyanide) [54] and to import iron into the cell (siderophore) [56,58]. The percentage of weathering genes did not differ significantly among the six glacial habitats (Table S13). Taxonomic classification of the microbiomes associated with rock-weathering genes in the different glacial habitats indicated that, at the class level, Betaproteobacteria were the most abundant taxa in ‘Sub’ and ‘Stream’ (Fig. 6a and Table S13). The second most abundant class was Gammaproteobacteria, with a higher abundance in ‘Snow’ (Fig. 6a and Table S13). At the genus level, *Pseudomonas* was the most abundant genus associated with rock-weathering processes; this genus was most abundant in ‘Snow’ (Fig. 6b and Table S14). *Polaromonas* was the second most abundant genus, with the highest abundance in ‘Sub’ (Fig. 6b and Table S14). Detailed information about relative abundances and multiple comparisons of the relative abundances of rock-weathering taxa at the class, genus and family levels among the glacial habitats are provided in Tables S13–S15. Furthermore, z-scores demonstrated that although taxa with rock-weathering genes were present in all habitats, the genera where these genes were most abundant were predominantly found in ‘Snow’ (Fig. 6). In ‘Soil’, mainly Flavobacteriia and Cyanophyceae had rock-weathering genes.

## Discussion

### Diversity, structure and taxonomic composition of functional genes among glacier habitats

The functional and taxonomic community structures of the microbiomes mostly differed between ‘Snow’ and the other habitats (except ‘Supra’; Table 1). The similarities between the fungal community in the ‘Snow’ and ‘Supra’ (higher presence of basidiomycetous yeasts such as *Leucosporidium* and *Rhodotorula* compared to other habitats) and the absence of snow-associated fungal taxa in the other glacial habitats (‘Ice’, ‘Sub’ and ‘Stream’) and in ‘Soil’ were also observed by Rime *et al*. [22]. Microbiomes in the air may be derived from soils, water and plants [78,79]; furthermore, taxa attached to dust particles [80] can be an important component of snow and supraglacial habitats but may be isolated from glacial habitats and outcompeted in soil habitats because of the ‘priority effect’ [22]. In addition, snow is the most diverse glacial habitat for microbiomes in terms of structure [81]. It is the only habitat that we studied that is not directly influenced by the glacial system and that is upstream of all glacial processes that shape glacial microbial communities [82]. These reasons may explain the significant difference in the functional and taxonomic community structure between ‘Snow’ and the other habitats in our study.

As for community composition, Pseudomonadota and Bacteroidota dominated the functional dataset in all glacial habitats. Their dominance has also been observed in ice, stream water and biofilms of glacier-fed streams in 26 Alpine glaciers using 454 pyrosequencing [83], ice and snow samples from Byron Glacier in Alaska [84] and glacial ice samples from Northern Schneeferner Glacier in Germany using metagenomic sequencing [85]. Their dominance in the functional dataset indicates that they may play important roles in nutrient cycling and metabolism in glacial habitats. For example, a link between the prevalence of cellulose and complex plant C degradation enzymes and the abundance of Bacteroidota has been confirmed [86]. In addition, we also found that Ascomycota was more abundant in the ‘Snow’ and ‘Supra’ in the ribosomal dataset, where these taxa may be derived from atmospheric deposition [22].

However, functional gene diversity surprisingly showed no significant differences across various glacial habitats, with the exception of richness in NCyc, which was highest in ‘Soil’ and lowest in both ‘Sub’ and ‘Supra’. Rime *et al*.’s investigation into the taxonomic diversity across different glacial and proglacial habitats of the Damma glacier indicated the highest levels of bacterial and fungal diversity in recently deglaciated soils [22]. The higher richness of taxa and N-cycling genes in proglacial soil habitats might be attributed to the development of a microbial community derived both from the soil and glacial environments, as bacteria in proglacial soils have already been shown to be originated from the ice, supra- and subglacial sediment and proglacial stream habitats [22]. In addition, the colonization of recently deglaciated barren soils by N-fixing microorganisms (e.g., Cyanobacteriota) may contribute to the higher abundance of N-cycling genes in proglacial soil habitats [87]. In fact, a higher abundance of Cyanobacteriota in ‘Soil’ samples was observed in the predicted gene taxonomy in our study (Figs 1 and S3 and Table S5).

### Shifts in functional pathways across glacial habitats

As glacial environments pose harsh life conditions to the survival of microbiomes, we especially focused on gene functions within the category of posttranslational modification, protein turnover and chaperones, which contains many functional genes related to stress responses (e.g., proteases and chaperones) [88,90]. Our results suggest that the relative gene abundances associated with this category were 1.9–3% across the six glacial habitats and were especially enriched in ‘Ice’ compared with ‘Snow’ (Fig. S13 and Table S16). These results support our second hypothesis and previous findings that microorganisms inhabiting glacial ice show unique adaptations to the cold, oligotrophic environment [2,4]. Cold-shock genes have already been found in sea-ice cryoconites using MinION metagenome sequencing [91]. In addition, metabolic adaptations linked to a psychrophilic lifestyle (such as the formation of cryoprotectants and maintenance of membrane fluidity) were identified in the glacial ice of the Northern Schneeferner [85]. The enrichment of these stress response genes may be a survival strategy for microbes in the extremely cold, low-nutrient glacial ice habitat [4].

We also found that genes related to cellular processes and signalling, such as signal transduction mechanisms [e.g. COG3434 (signal transduction protein), COG3026 (sigma E regulatory protein) and COG3073 (anti-sigma-E protein RseA)]; intracellular trafficking, secretion and vesicular transport [e.g., COG5314 (conjugal transfer protein Trbj), COG4970 (type II transport protein GspH) and COG4968 (type IV fimbrial biogenesis transmembrane protein)] and cell motility [e.g., COG1580 (flagellar basal body-associated protein), COG1677 (flagellar hook-basal body complex protein FliE) and COG3144 (flagellar hook-length control protein)], were enriched in the ‘Sub’. Meltwater from supraglacial ice supplies oxygen to the subglacial environment, creating gradients of redox conditions in the subglacial habitats [4,92]. This alteration in redox conditions within the subglacial habitat can lead to a high abundance of genes associated with signal transduction, which are involved in rapid responses to environmental stimuli [93,94].

### Shifts in the microbial genetic potential of C-degrading genes among habitats

Although the DOC content was highest in the ‘Supra’, ‘Sub’ and ‘Soil’ (Table S1), the abundance of functional genes related to labile C degradation [such as glycoside hydrolases (GHs)] was enriched in ‘Soil’ and ‘Snow’ (Table 2). This result contradicts our third hypothesis and suggests that higher DOC contents were not always correlated with higher abundances of genes related to labile C degradation, possibly because both labile and recalcitrant DOCs exist in glacial habitats [95]. In addition, we found that genes related to PL (mainly responsible for degrading pectin and cellulose) were more abundant in ‘Soil’ than in ‘Snow’. This plant-derived biomass (pectin and cellulose) in deglaciated soils presumably originated from beneath the glacier [96] or from the glacier surface. In addition, genes related to AAs, which are associated with refractory lignin degradation, were more abundant in ‘Sub’ (Table 2). Findings from a previous study also suggested that heterotrophs can utilize ancient recalcitrant C in subglacial sediment, which may have been deposited in preglacial times [96,97]. We conclude that although the DOC content in ‘Sub’ was high, the ancient recalcitrant C stored there resulted in abundant microbial functional genes related to refractory C degradation.

Moreover, we found that the most abundant C-degrading genes annotated using CAZy in the Damma glacial habitats were glycosyl transferases (GTs) and glycoside hydrolases (GHs). The dominance of GHs and GTs in CAZymes has also been observed in Tsomgo Lake in the Eastern Himalayas [98] and Teesta River in Sikkim Himalayas [99]. GT catalyses the transfer of sugar moieties to form glycosidic bonds, and GH is a widespread group of enzymes that can hydrolyse the glycosidic bond between carbohydrates [100]. Their high abundance in the habitats considered here indicates that genes related to GHs and GTs may play major roles in the synthesis and degradation of carbohydrates in glacial habitats. Overall, our results on C degradation genes suggest that genes related to labile C degradation in ‘Soil’, ‘Supra’ and ‘Sub’ were not always more abundant than in other habitats, though the DOC content was higher in these habitats, and genes related to recalcitrant C degradation were enriched in ‘Sub’. With glacier retreat, a shift in the dynamics between these different glacial habitats, and the associated functional potential microbial functionality (e.g., organic C and C-degrading genes), might impact the role of the glacial biome in nutrient and C cycling; this could also influence the amount of organic C that is stored in glacial ecosystems and that can be released to the downstream ecosystem [101,103]. A change in this export to downstream ecosystems could lead to a change in ecosystem services, such as nutrient cycling [101,104, 105] and food web structure [106].

### Shifts in the microbial genetic potential for N-cycling across glacial habitats

Organic degradation and synthesis (OD&S) was the most abundant N pathway in the glacial habitats, followed by gene families ascribed to nitrate reduction [ANR, denitrification and dissimilatory nitrate reduction (DNR)], indicating that the organic transformation of N and nitrate reduction may be the main N transformation processes in glacial habitats. As far as we know, there are no comparable studies related to N pathways in glacier habitats from the European Alps. The dominance of genes related to OD&S in N cycling processes has also been found in high-alpine soils [88,107, 108].

However, contradicted with our fourth hypothesis, genes related to nitrate reduction (ANR, denitrification and DNR) were not significantly enriched in ‘Sub’ (Table 2). Nevertheless, a high relative abundance of denitrification and DNR in ‘Sub’ compared with other glacial habitats was found in our study (Fig. S15), which agrees with other studies. For instance, a high abundance of genes related to denitrification and DNR has been found in subglacial sediment from East Antarctica [109] and an Alaskan glacier [29] using metagenomic sequencing. In addition, N-cycling genes related to ANR were also enriched in ‘Ice’, which suggested that the microbiome in basal glacial ice uses inorganic nitrogen more as an N source for biosynthesis than as an energy source [85].

In our dataset, genes related to N_2_ fixation were enriched in the ‘Soil’ habitat (Table 2), most of which were taxonomically assigned to Cyanobacteriota (Table S17). Cyanobacterial sequences have also been retrieved from barren soils near the Damma glacier [87], which may have contributed to the enrichment of N_2_-fixing genes. In addition, the DOC content is much higher in soil habitats, the N_2_ fixation process is highly energy demanding [110] and the high levels of available C in soil habitat may provide microbes with sufficient energy for biological N_2_ fixation. With global warming, a large amount of nitrogen has been exported from the Alpine glacier, influencing the phytoplankton diversity of productivity in Alpine lakes [111,112]. Our study confirms the high abundance of organisms involved in N-cycling in glacial ecosystems; these organisms can be exported to downstream habitats and impact their dynamics.

### Differences in weathering genes among glacial habitats

In support of our last hypothesis, weathering genes were found in all the glacial habitats, especially in ‘Snow’. The snow microbiome contained many Pseudomonadota, which are known to be active in weathering [53,56, 113]. In addition, under iron-depleted environments, iron-containing mineral dust has been shown to enhance snow algal development, and snow algal microbial communities can improve mineral dissolution [114]. Snow microorganisms can also colonize Arctic soils after snow melt [115]. Our results suggest that the accelerated rock weathering with snow cover, which has also been documented previously [116,118], may result not only from chemical weathering by increased snow-derived moisture but also from biological weathering carried out by snow-dwelling microbes (e.g. Gammaproteobacteria). The most abundant rock-weathering genus, *Pseudomonas* (affiliated with class Gammaproteobacteria), was most abundant in the ‘Snow’. *Pseudomonas* has been reported to be able to degrade granite in the Damma glacier site [113]. The high abundance of *Pseudomonas* in the ‘Snow’ indicates its importance of rock weathering in snow-buried soils in glacial ecosystems.

Microbially mediated rock weathering has been widely reported in subglacial sediments [37,119,121]. *Polaromonas* (Betaproteobacteria), which was the most abundant rock-weathering genus in the ‘Sub’, has been shown to be significantly associated with mineral weathering in the Damma glacier forefield [56]. The high abundance of *Polaromonas* in ‘Sub’ suggests that it may play an important role in the weathering of bedrock minerals. This in turn can influence subglacial water chemistry [17,122], nutrient cycling (e.g. sulphide and iron oxidation) [15,18, 37, 123] and C fixation [124], as microbiomes in subglacial environments are isolated from sunlight and direct input from the outside environment and thus rely on metabolic substrates and nutrients released from rock weathering [17]. In addition, subglacial rock weathering can be the source of element (e.g. iron) cycling in downstream water ecosystems [125]. Our study highlights the potential for extracting information regarding rock weathering using metagenomic data. With the accelerating pace of global warming, rock-weathering microbes may play increasingly important roles in the nutrient accumulation of glacial and adjacent ecosystems. Therefore, the identification and isolation of rock-weathering microbes is of pivotal importance.

## Conclusion

Our shotgun metagenomic study demonstrates that the microbial communities present in the Damma glacier ecosystem harbour a high microbial functional potential for several C and N metabolic pathways. Microbiomes from different glacial habitats varied in their functional genetic potential, highlighting the need for more comparative studies among the different glacial compartments. Generally, microbiomes in the ‘Ice’ harboured more genes related to stress responses and assimilatory nitrate reduction, suggesting that ice microbiomes are more stress-tolerant than other glacial habitats and that assimilation of inorganic N sources is important for the microbial biosynthesis in glacial ice. The microbiomes in ‘Sub’ were enriched in genes related to the degradation of recalcitrant C, which may have been deposited in preglacial times, and in genes involved in cellular processes and signalling, which might be attributed to diverse chemolithotrophic processes under dark anoxic conditions with high concentrations of iron and sulphur. Our results also suggest that proglacial soil habitats are enriched in labile C-degrading and N_2_-fixing genes, indicative of a community with a more favourable nutrient supply. Our results further identified the presence of rock-weathering microbes in glacial habitats, especially in snow and subglacial habitats, with *Pseudomonas* and *Polaromonas* being the two most abundant genera involved in rock weathering, suggesting their important role in mineral weathering and nutrient cycling in glacial ecosystems. These results represent a further step towards a more comprehensive understanding of the distribution of microbial functional genes in glacial habitats. Moreover, they may help to predict the changes in glacial microbial functions and potentially also their influence on downstream ecosystems under accelerating climate change. Finally, our results provide valuable information for achieving targeted biodiversity monitoring and better ecological protection of glacial habitats.

## supplementary material

10.1099/mgen.0.001301 Uncited Supplementary Material 1.

10.1099/mgen.0.001301 Uncited Supplementary Material 2.

## Abbreviations

AAs: auxiliary activities
ANR: assimilatory nitrate reduction
DNR: dissimilatory nitrate reduction
GHs: glycoside hydrolases
GTs: glycosyl transferases
LSD: least significant difference
OD&S: organic degradation and synthesis
OGs: orthologous groups
PCoA: principal coordinate analysis
PLs: polysaccharide lyases
SSU rRNA: small subunit ribosomal RNA
