# Supplementary material for: Microbial genetic potential differs among cryospheric habitats of the Damma glacier
Source: Microb Genom. 2024 Oct 1;10(10):001301. doi: 10.1099/mgen.0.001301 (PMC11443553; doi:10.1099/mgen.0.001301)
Supplement: Uncited Supplementary Material 1. [file mgen-10-01301-s001.pdf]

Supplemental Material

Microbial genetic potential differs among cryospheric habitats of the Damma glacier

Maomao Feng, Serina Robinson, Weihong Qi, Arwyn Edwards, Beat Stierli, Marcel Van der Heijden, Beat Frey, Gilda Varliero

Supplementary Results

Shifts in eggNOG genes across different habitats

Genes involved in category replication, recombination and repair were most abundant (4.9–8.4% across all habitats), followed by those involved in cell wall/membrane/envelope biogenesis (4.3–7.0%), amino acid transport and metabolism (3.3–5.8%), energy production and conversion (2.6–5.2%), and inorganic ion transport and metabolism (2.8–5.5%; Fig. S13).

Changes in C-degrading genes across different habitats

Among all the C-degrading genes annotated using CAZy, glycoside hydrolases (0.88–1.53%; hydrolysis and/or rearrangement of glycosidic bonds, can use substrates such as cellulose, starch and hemicellulose) was the most abundant enzyme class, followed by glycosyltransferases (0.77–1.34%, responsible for forming glycosidic bonds), carbohydrate-binding modules (CBMs; 0.43–0.62%, adhesion to carbohydrates), carbohydrate esterases (0.11–0.18%, hydrolysis of carbohydrate esters), polysaccharide lyases (0.04–0.11%, non-hydrolytic cleavage of glycosidic bonds, can degrade starch and pectin) and auxiliary activities (0.03–0.10%, responsible for plant cell-wall degradation [1](Fig. S14).

Changes in N-cycling genes across different habitats

The most abundant N-cycling family was the one involved in organic degradation and synthesis, which was between 0.29% and 0.36% in all habitats, followed by those involved in denitrification (0.02–0.08%), assimilatory nitrate reduction (0.02–0.08%), and dissimilatory nitrate reduction (0.18–0.03%). Genes involved in N fixation, nitrification and anammox were less abundant (Fig. S15).

1. **Levasseur A, Drula E, Lombard V, Coutinho PM, Henrissat B**. Expansion of the enzymatic repertoire of the CAZy database to integrate auxiliary redox enzymes. *Biotechnology for Biofuels* 2013;6(1):41.

Supplementary Tables

Microbial genetic potential differs among cryospheric habitats of the Damma glacier

Maomao Feng, Serina Robinson, Weihong Qi, Arwyn Edwards, Beat Stierli, Marcel Van der Heijden, Beat Frey, Gilda Varliero

Table S1. Biotic and abiotic characteristics of the six Damma glacial habitats. Significance was analyzed by one-way analysis of variance (ANOVA) followed by a least significant difference (LSD) test. *P*-values were corrected for multiple testing with the Benjamini–Hochberg method. Values are means ± SD, followed by bold lowercase letters indicating significant pairwise differences ($P < 0.05$). Habitats that share the same letter are not significantly different, while those with different letters are significantly different. Pairwise significant differences between habitats are annotated by different lowercase letters in bold. The data are derived from Rime, Hartmann [1].

	**Snow**	**Stream**	**Ice**	**Sub**	**Supra**	**Soil**
**Soil geochemistry**						
pH$_{[H2O]}$	6.2±0.1**ab**	6.45±0.1**a**	5.8±0.2**bc**	5.4±0.2**d**	6.5±0.2**a**	5.4±0.1**cd**
DOC (ug g^{-1} soil)	0.7±0.1**bc**	0.003±0.006**c**	1.0±1.0**bc**	12.5±8.4**ab**	10.6±0.2**abc**	17.1±5.5**a**
DON (ug g^{-1} soil)	0.10±0.06**c**	0.19±0.05**bc**	0.08±0.07**c**	0.80±0.15**ab**	0.51±0.02**abc**	1.05±0.48**a**
Cl^- (ug g^{-1} soil)	0.07±0.03**b**	0.05±0.02**b**	0.05±0.03**b**	1.93±0.21**a**	0.18±0.03**b**	0.29±0.02**b**
NO_2^- (ug g^{-1} soil)	0.01±0**a**	0.01±0**a**	0.01±0**a**	0.01±0**a**	0.01±0**a**	0.01±0**a**
NO_3^- (ug g^{-1} soil)	0.12±0.09**b**	0.45±0.06**a**	0.11±0.02**b**	0.21±0.10**b**	0.05±0.01**b**	0.06±0.01**b**
PO_4^{3-} (ug g^{-1} soil)	0.12±0**a**	0.12±0**a**	0.12±0**a**	0.12±0**a**	0.12±0**a**	0.12±0**a**
SO_4^{2-} (ug g^{-1} soil)	0.06±0.02**c**	0.40±0.06**ab**	0.05±0.01**c**	0.29±0.16**b**	0.31±0.02**b**	0.54±0.04**a**
NH_4^+ (ug g^{-1} soil)	0.08±0.07**b**	0.02 ±0.00**b**	0.03±0.00**b**	0.41 ±0.18**ab**	0.42±0.14**ab**	0.82 ±0.48**a**
**16S and ITS gene copy numbers**						
16S	(2.0±**0.6) ×10**^{3}**c**	(1.5±**0.4) ×10**^{3}**c**	(4.4±**6.7) ×10**^{2}**c**	(1.6±**0.3) ×10**^{6}**b**	(1.5±**0.2) ×10**^{6}**b**	(3.3±**0.6) ×10**^{6}**a**
ITS	(7.9±**1.2) ×10**^{3}**a**	89±**6.5a**	5.7±**4.6a**	(1.0±**0.6) ×10**^{3}**a**	(1.6±**1.2) ×10**^{5}**a**	(1.1±**0.5) ×10**^{4}**a**
16S/ITS	0.25±**0.04b**	16.6±**4.2b**	49.0±**53.4b**	177.4±**74.3a**	13.1±**6.9b**	37.7±**21.7b**

pH$_{[H2O]}$, pH measured in water; DOC, dissolved organic carbon; DON, dissolved organic nitrogen; Cl^-, chloride; NO_2^-: nitrite; NO_3^-, nitrate; PO_4^{3-}, phosphate; SO_4^{2-}, sulphate; NH_4^+, ammonium; 16S: copy numbers of bacterial 16S gene; ITS: copy numbers of fungal ITS region. 16S/ITS: Bacterial and fungal gene copy number ratio

Snow: surface snow; Stream: proglacial stream water; Ice: subglacial ice; Sub: subglacial sediment; Supra: supraglacial sediment; Soil: recently deglaciated soil.

1. **Rime T, Hartmann M, Frey B**. Potential sources of microbial colonizers in an initial soil ecosystem after retreat of an alpine glacier. *ISME J* 2016;10(7):1625-1641

Table S2. Read content per sample. All counts are reported as the sum of both forward and reverse reads. The sample IDs consist of the habitat followed by the replicate number (1, 2 or 3). Raw reads: total number of sequences after sequencing; HQ reads: high-quality sequences after quality control; Snow: surface snow; Stream: proglacial stream water; Ice: subglacial ice; Sub: subglacial sediment; Supra: supraglacial sediment; Soil: recently deglaciated soil.

Sample	Raw reads	No. HQ reads	% HQ reads
Snow_1	26,464,440	26,301,220	99.4
Snow_2	24,969,600	24,803,927	99.3
Snow_3	13,881,896	13,809,312	99.5
Stream_1	21,123,634	21,013,397	99.5
Stream_2	19,287,708	19,197,673	99.5
Stream_3	34,668,330	34,517,135	99.6
Ice_1	17,324,194	17,239,239	99.5
Ice_2	17,485,802	17,371,598	99.3
Ice_3	7,904	5,300	67.0
Sub_1	12,768,972	12,650,194	99.1
Sub_2	21,474,762	21,339,619	99.4
Sub_3	20,950,932	20,800,880	99.3
Supra_1	15,686,304	15,614,283	99.5
Supra_2	7,418,398	7,355,598	99.2
Supra_3	28,852,186	28,744,352	99.6
Soil_1	12,613,100	12,500,206	99.1
Soil_2	13,019,150	12,941,984	99.4
Soil_3	18,654,274	18,555,386	99.5

Table S3. Total number of sequences and the percentage of protein-coding genes and contigs in the six Damma glacial habitats. Significance was analyzed by one-way analysis of variance (ANOVA) followed by a least significant difference (LSD) test. *P*-values were corrected for multiple testing with the Benjamini–Hochberg method. Values are mean ± SD (n=3, except for "Ice" where n=2). Significant differences between habitats are annotated by different lowercase letters in bold. Habitats that share the same letter are not significantly different, while those with different letters are significantly different. Raw reads: total number of sequences after sequencing; HQ reads: high-quality sequences after quality control; CDSs: coding DNA sequences; Snow: surface snow; Stream: proglacial stream water; Ice: subglacial ice; Sub: subglacial sediment; Supra: supraglacial sediment; Soil: recently deglaciated soil.

	**Snow**	**Stream**	**Ice**	**Sub**	**Supra**	**Soil**
Raw reads (×10^6)	21.8±6.9**a**	25.0±8.4**a**	17.4**a**	18.4±4.9**a**	17.3±10.8**a**	14.8±3.4**a**
HQ reads (×10^6)	21.7±6.8**a**	24.9±8.4**a**	17.3**a**	18.3±4.9**a**	17.2±10.8**a**	14.7±3.4**a**
No. reads mapped to CDSs (×10^6)	8.9±4.0**a**	9.4±3.6**a**	11.0**a**	13.6±3.6**a**	10.9±8.4**a**	3.2±1.6**a**
% Reads mapped to CDSs	40.0±6.8**bc**	39.1±16.4**bc**	63.9**ab**	74.8±1.3**a**	58.0±15.2**ab**	21.3±6.7**c**
No. reads mapped to contigs (×10^6)	13.5±4.1**a**	18.9±6.0**a**	13.8**a**	17.4±4.6**a**	16.0±9.2**a**	5.2±2.0**a**
% Reads mapped to contigs	63.2±1.8**b**	77.1±15.0**ab**	80.0**ab**	96.0±0.5**a**	95.6±5.3**a**	35.3±6.6**c**

Table S4. Overall assembly statistics of the metagenomic data.

Assembly statistic	Value
Number of contigs	2,443,106
Assembly size (bp)	1,960,450,729
Mean contig length (bp)	802
Median contig length (bp)	8,007
Maximum contig length (bp)	419,451
Minimum contig length (bp)	200
N50 (bp)	1,000
GC content (%)	46
Number of predicted genes	3,337,869
Number of predicted genes annotated with CAZy	33,369
Number of pPredicted genes annotated with NCyc	5,251
Number of predicted genes annotated with eggNOG	1,595,443

Table S5. Comparison of the relative abundances of major taxonomic phyla (with an average relative abundance > 1% in at least one habitat) in all predicted, functional (annotated using eggNOG, CAZy and NCyc), and ribosomal (SSU) genes among different Damma glacial habitats. Values are means ± SD (n=3, except for "Ice" where n=2). Snow: surface snow; Stream: proglacial stream water; Ice: subglacial ice; Sub: subglacial sediment; Supra: supraglacial sediment; Soil: recently deglaciated soil. Significant differences are annotated with different lowercase letters after the relative abundances. Habitats that share the same letter are not significantly different, while those with different letters are significantly different. For Table S5, please refer to Table.S5.xlsx.

Table S6. Changes in alpha-diversity for functional (annotated using eggNOG, CAZy and NCyc database) and small subunit ribosomal RNA (SSU rRNA) genes of microbiomes in the six Damma glacial habitats. Significance was tested by one-way analysis of variance (ANOVA) and least significant difference (LSD) tests. ANOVA $P < 0.05$ are reported in bold. LSD P were corrected for multiple testing with the Benjamini–Hochberg method, and values are reported as means ± standard deviation (SD) (except for Ice where values are reported as means). Significant (P<0.05) differences based on the LSD test are annotated by different lowercase letters in bold. Habitats that share the same letter are not significantly different, while those with different letters are significantly different. Snow: surface snow; Stream: proglacial stream water; Ice: subglacial ice; Sub: subglacial sediment; Supra: supraglacial sediment; Soil: recently deglaciated soil. F: ratio that compares the variance between group means to the variance within groups in ANOVA.

	**eggNOG**				**CAZy**				**NCyc**				**SSU rRNA**			
	Richness		Shannon		Richness		Shannon		Richness		Shannon		Richness		Shannon	
**ANOVA**																
	F	*P*	*F*	*P*	*F*	*P*	*F*	*P*	*F*	*P*	*F*	*P*	*F*	*P*	*F*	*P*
Habitat	2.4	0.10	1.7	0.2	4.0	**0.03**	2.4	0.1	6.3	**0.005**	3.6	**0.03**	5.4	**0.01**	2.5	0.09
**LSD**																
Snow	1635±34**a**		6.6±0.1**a**		3469±555**a**		6.8±0.6**a**		483±62**ab**		4.9±0.5**a**		754±122**ab**		5.3±0.4**a**	
Stream	1566±95**a**		6.4±0.1**a**		3614±1561**a**		6.9±0.6**a**		466±171**ab**		4.7±0.2**a**		634±204**ab**		4.6±0.8**a**	
Ice	1568**a**		6.5**a**		3522**a**		7.1**a**		584**ab**		5.3**a**		452**ab**		3.8**a**	
Sub	1454±39**a**		6.4±0.1**a**		1875±283**a**		5.9±0.4**a**		294±47**b**		4.1±0.7**a**		256±158**b**		2.4±1.8**a**	
Supra	1305±281**a**		6.0±0.7**a**		1531±1440**a**		5.2±1.7**a**		228±163**b**		3.5±1.2**a**		387±298**ab**		3.6±2.0**a**	
Soil	1544±7 **a**		6.5±0.02**a**		4353±267**a**		7.0±0.4**a**		659±72**a**		5.5±0.2**a**		944±152**a**		5.5±0.9**a**	

Table S7. Comparison of the beta dispersion of soil functional (annotated using eggNOG, CAZy and NCyc) and small subunit ribosomal RNA (SSU rRNA) genes in the six Damma glacial habitats. Snow: surface snow; Stream: proglacial stream water; Ice: subglacial ice; Sub: subglacial sediment; Supra: supraglacial sediment; Soil: recently deglaciated soil. "t": the magnitude of difference in dispersions between groups. "*P*": probability that the observed differences in dispersion occurred by chance.

	**eggNOG**		**CAZy**		**NCyc**		**SSU rRNA**	
	t	*P*	*t*	*P*	*t*	*P*	*t*	*P*
Snow vs. Stream	1.10	0.560	0.57	1	0.95	0.62	1.42	0.48
Snow vs. Ice	2.15	0.42	2.11	0.39	1.97	0.40	0.49	1
Snow vs. Sub	2.48	0.11	3.90	0.11	3.12	0.08	3.11	0.10
Snow vs. Supra	4.34	0.10	4.18	0.10	5.22	0.1	5.78	0.12
Snow vs. Soil	0.63	0.61	1.20	0.63	0.52	0.57	0.45	0.58
Stream vs. Ice	1.75	0.61	1.26	1	1.64	0.60	1.41	1
Stream vs. Sub	0.09	1	0.96	0.40	0.34	1	0.99	0.58
Stream vs. Supra	2.25	0.50	1.77	0.28	2.06	0.30	2.83	0.10
Stream vs. Soil	1.41	0.47	1.22	0.48	1.23	0.49	1.70	0.51
Ice vs. Sub	8.38	0.21	13.49	0.22	16.71	0.10	3.01	0.34
Ice vs. Supra	4.63	0.28	5.69	0.28	7.25	0.201	5.38	0.30
Ice vs. Soil	0.94	1	0.33	1	1.01	1	0.08	1
Sub vs. Supra	3.43	0.09	1.96	0.10	4.01	0.09	2.10	0.3
Sub vs. Soil	2.61	0.21	4.20	0.10	3.11	0.10	3.55	0.10
Supra vs. Soil	4.45	0.10	4.60	0.10	5.10	0.10	6.40	0.10

Table S8. Soil geochemistry and 16S/ITS ratio correlations with microbial functional (annotated using eggNOG, CAZy and NCyc databases) and small subunit ribosomal RNA (SSU rRNA) gene structure in the six Damma glacial habitats. The results were obtained from "Mantel" function in R. Significant values are marked in (P<0.05). R^2: the proportion of variance in microbial functional community structure that is explained by the variance in the environmental variables; P: significance of the correlation between microbial functional and SSU ribosomal gene structure and environmental variables.

**Variables**	**Predicted**		**eggNOG**		**CAZy**		**NCyc**		**SSU rRNA**	
	R^2	P	R^2	P	R^2	P	R^2	P	R^2	P
pH	0.27	0.12	0.26	0.13	0.28	0.11	0.23	0.17	0.27	0.11
DOC	0.22	0.16	0.21	0.21	0.22	0.18	0.20	0.22	0.10	0.45
DON	0.24	0.15	0.23	0.17	0.24	0.15	0.21	0.19	0.14	0.36
Cl^-	0.31	0.08	0.33	0.06	0.33	0.07	0.31	0.07	**0.60**	**0.00**
NO_2^-	0.00	1.00	0.00	1.00	0.00	1.00	0.00	1.00	0.00	1.00
NO_3^-	0.03	0.80	0.04	0.78	0.04	0.80	0.05	0.74	0.18	0.24
PO_4^{3-}	0.00	1.00	0.00	1.00	0.00	1.00	0.00	1.00	0.00	1.00
SO_4^{2-}	**0.62**	**0.00**	**0.60**	**0.00**	**0.61**	**0.00**	**0.56**	**0.00**	**0.55**	**0.00**
NH_4^+	0.17	0.28	0.15	0.30	0.16	0.30	0.13	0.37	0.04	0.77
16S	0.29	0.08	0.27	0.12	0.28	0.09	0.24	0.14	0.10	0.49
ITS	0.16	0.30	0.16	0.30	0.18	0.24	0.15	0.33	0.05	0.73
16S/ITS	**0.44**	**0.02**	**0.45**	**0.01**	**0.46**	**0.01**	**0.41**	**0.02**	**0.62**	**0.00**

pH, pH measured in water; DOC, dissolved organic carbon; DON, dissolved organic nitrogen; Cl^-, chloride; NO_2^-: nitrite; NO_3^-, nitrate; PO_4^{3-}, phosphate; SO_4^{2-}, sulphate; NH_4^+, ammonium; 16S: copy numbers of bacterial 16S gene; ITS: copy numbers of fungal ITS region. 16S/ITS: Bacterial and fungal gene copy number ratio

Table S9. Numbers of functional genes annotated using eggNOG (only COGs were included), CAZy and NCyc databases that represent differentially abundant genes (DAGs) between each two Damma glacial habitats. For example, in the "Stream vs. Snow" column, "Up" means the number of genes that were more abundant in Stream than in Snow, while "Down" means the number of genes that were more abundant in Snow than in Stream. Total: total number of significantly differentially abundant genes; DAGs: genes that were significantly (*P*<0.05) differentially abundant between two habitats; Up: number of overrepresented genes; Down: number of underrepresented genes; Snow: surface snow; Stream: proglacial stream water; Ice: subglacial ice; Sub: subglacial sediment; Supra: supraglacial sediment; Soil: recently deglaciated soil.

		**Snow vs. Stream**	**Snow vs. Ice**	**Snow vs. Sub**	**Snow vs. Supra**	**Snow vs. Soil**	**Stream vs. Ice**	**Stream vs. Sub**	**Stream vs. Supra**	**Stream vs. Soil**	**Ice vs. Sub**	**Ice vs. Supra**	**Ice vs. Soil**	**Sub vs. Supra**	**Sub vs. Soil**	**Supra vs. Soil**
Eggnog	Total	1931	1931	1931	1931	1931	1931	1931	1931	1931	1931	1931	1931	1931	1931	1931
	DAGs	406	851	406	313	690	139	188	104	99	400	194	186	120	466	152
	Up	171	419	171	110	260	60	88	45	33	237	110	61	76	176	57
	Down	235	432	235	203	430	79	100	59	66	163	84	125	44	290	95
CAZy	Total	12726	12726	12726	12726	12726	12726	12726	12726	12726	12726	12726	12726	12726	12726	12726
	DAGs	1719	3495	2371	1373	2195	250	134	30	442	76	426	452	150	1712	494
	Up	636	1117	410	169	1360	59	12	23	255	68	191	340	139	1574	466
	Down	1083	2378	1961	1204	835	191	122	7	187	8	235	112	11	138	28
NCyc	Total	2145	2145	2145	2145	2145	2145	2145	2145	2145	2145	2145	2145	2145	2145	2145
	DAGs	272	471	340	176	319	67	34	9	26	80	52	98	30	166	42
	Up	67	198	58	17	147	15	2	3	9	46	28	45	28	148	40
	Down	205	273	282	159	172	52	32	6	17	34	24	53	2	18	2

Table S10. Full list of the functional genes annotated with the eggNOG database (only clusters of orthologous groups [COGs] were included) that are differentially abundant ($P < 0.05$ and $|LFC| > 2$) between the pairwise comparisons. For Table S10, please refer to "Table S10 deseq-result_eggNOG.xlsx".

Table S11. Full list of the C-cycling genes annotated with the CAZy database that are differentially abundant ($P < 0.05$ and $|LFC| > 7$) between the pairwise comparisons. For Table S11, please refer to "Table S11 deseq-result_cazy.xlsx".

Table S12. Full list of the N-cycling genes annotated with the NCyc database that are differentially abundant ($P < 0.05$ and $|LFC| > 3$) between the pairwise comparisons. For Table S11 please refer to "Table S12 deseq-result_ncyc.xlsx".

Table S13. The relative abundances (%) of total weathering genes and taxa involved in rock weathering at the class level (top 10) in different Damma glacial habitats. Significances were analyzed by one-way analysis of variance (ANOVA) followed by a least significant difference (LSD) test. *P*-values were adjusted using the Benjamini–Hochberg method. Significant differences are annotated by different lowercase letters in bold. Habitats that share the same letter are not significantly different, while those with different letters are significantly different. Values are means ± SD (n=3, except for "Ice" where n=2) followed by lowercase letters indicating significant differences ($P < 0.05$). Snow: surface snow; Stream: proglacial stream water; Ice: subglacial ice; Sub: subglacial sediment; Supra: supraglacial sediment; Soil: recently deglaciated soil.

Class	Habitat					
	Snow	Stream	Ice	Sub	Supra	Soil
Betaproteobacteria	0.087005 ± 0.015929**a**	0.141842 ± 0.175453**a**	0.022125**a**	0.191583 ± 0.061193**a**	0.080881 ± 0.048743**a**	0.041006 ± 0.023258**a**
Gammaproteobacteria	0.180943 ± 0.153375**a**	0.004307 ± 0.000183**a**	0.006532 **a**	0.013913 ± 0.015311**a**	0.002007 ± 0.001287**a**	0.017836 ± 0.009712**a**
Chitinophagia	0.007277 ± 0.003126**a**	0.009685 ± 0.007684**a**	0.006556 **a**	0.004489 ± 0.003029**a**	0.024439 ± 0.028928**a**	0.014208 ± 0.00054**a**
Sphingobacteriia	0.025196 ± 0.015752**a**	0.006694 ± 0.004749**ab**	0.001424 **b**	0.000983 ± 0.000491**b**	0.002961 ± 0.002748**b**	0.010715 ± 0.001346**ab**
Alphaproteobacteria	0.00925 ± 0.007079**a**	0.002672 ± 0.000659**a**	0.00494 **a**	0.004264 ± 0.003098**a**	0.001937 ± 0.00199**a**	0.013237 ± 0.00978**a**
Cytophagia	0.016547 ± 0.001058**a**	0.00481 ± 0.003628**b**	0.001679 **b**	0.000198 ± 0.000141**b**	0.006431 ± 0.005808**b**	0.00342 ± 0.000868**b**
Actinomycetia	0.012413 ± 0.013767**a**	0.003646 ± 0.004449**a**	0.000756 **a**	0.000552 ± 0.000426**a**	0.003092 ± 0.004755**a**	0.002399 ± 0.001231**a**
Flavobacteriia	0.001293 ± 0.000456**a**	0.006624 ± 0.004925**a**	0.004278 **a**	0.0025 ± 0.003761**a**	0.000653 ± 0.000987**a**	0.005657 ± 0.001872**a**
Cyanophyceae	0.000507 ± 0.000122**a**	0.002001 ± 0.000928**a**	0.002999 **a**	8e-06 ± 7e-06**a**	2.9e-05 ± 2.7e-05**a**	0.009183 ± 0.007608**a**
Bacilli	2.7e-05 ± 2.9e-05**a**	0.000922 ± 7.9e-05**a**	0.000536 **a**	0.001096 ± 0.000976**a**	0.000743 ± 0.001181**a**	0.000311 ± 9.8e-05**a**
Total weathering genes	0.34998 ± 0.166903**a**	0.192714 ± 0.160768**a**	0.075516 **a**	0.22809 ± 0.044427**a**	0.130007 ± 0.035167**a**	0.13298 ± 0.042691**a**

Table S14. The relative abundances (%) of taxa involved in rock weathering at the genus level (top 20) in different Damma glacial habitats. Significances were analyzed by one-way analysis of variance (ANOVA) followed by a least significant difference (LSD) test. *P*-values were adjusted using the Benjamini–Hochberg method. Significant differences are annotated by different lowercase letters in bold. Habitats that share the same letter are not significantly different, while those with different letters are significantly different. Values are means ± SD (n=3, except for "Ice" where n=2) followed by lowercase letters indicating significant differences ($P < 0.05$). Snow: surface snow; Ice: subglacial ice; Stream: proglacial stream water; Sub: subglacial sediment; Supra: supraglacial sediment; Soil: recently deglaciated soil.

Genus	Habitat					
	Snow	Stream	Ice	Sub	Supra	Soil
Pseudomonas	0.178548 ± 0.15182**a**	0.001925 ± 0.001082**a**	0.000918 **a**	0.003997 ± 0.006295**a**	0.00023 ± 0.000143**a**	0.008881 ± 0.010511**a**
Polaromonas	0.001171 ± 0.000504**b**	0.003798 ± 0.002967**b**	0.004813 **b**	0.095221 ± 0.062303**a**	0.00538 ± 0.003478**b**	0.005296 ± 0.004569**b**
Methylotenera	0.000223 ± 4.9e-05**a**	0.00576 ± 0.00445**a**	0.000347 **a**	0.002611 ± 0.003064**a**	0.043819 ± 0.040489**a**	0.007836 ± 0.001366**a**
Zoogloea	3e-06 ± 6e-06**a**	3.3e-05 ± 2.3e-05**a**	0.000143 **a**	0.027649 ± 0.040417**a**	6.6e-05 ± 8.4e-05**a**	0.000122 ± 0.000148**a**
Massilia	0.018358 ± 0.010352**a**	0.000552 ± 0.00046**b**	0.000964 **b**	0.004049 ± 0.002285**b**	0.000108 ± 9.4e-05**b**	0.001254 ± 0.001276**b**
Janthinobacterium	0.017156 ± 0.003519**a**	0.000492 ± 0.000375**b**	0.00094 **b**	0.001271 ± 0.001651**b**	0.000312 ± 0.000273**b**	0.002233 ± 0.002497**b**
Paraflavitalea	0.000407 ± 0.000371**a**	0.00034 ± 0.000263**a**	0.000534 **a**	0.000515 ± 0.000578**a**	0.01961 ± 0.032992**a**	0.000177 ± 2.1e-05**a**
Undibacterium	0.002213 ± 0.002049**a**	0.001381 ± 0.00032**a**	0.000129 **a**	0.0132 ± 0.021681**a**	0.000768 ± 0.000729**a**	0.001424 ± 0.000279**a**
Mucilaginibacter	0.010977 ± 0.014053**a**	0.001927 ± 0.001483**a**	0.000235 **a**	8.3e-05 ± 0.000104**a**	0.000479 ± 0.000797**a**	0.004738 ± 0.002164**a**
Glaciimonas	0.003549 ± 0.003126**a**	0.006967 ± 0.004519**a**	0.002725 **a**	0.002159 ± 0.001788**a**	0.001481 ± 0.001831**a**	0.001897 ± 0.001393**a**
Noviherbaspirillum	0.000958 ± 0.000412**a**	0.000634 ± 0.000441**a**	0.000936 **a**	0.012491 ± 0.01217**a**	0.000569 ± 0.000587**a**	0.001669 ± 0.001851**a**
Hymenobacter	0.013535 ± 0.000745**a**	0.000544 ± 0.000227**b**	0.000235 **b**	1.7e-05 ± 4e-06**b**	0.000435 ± 0.000372**b**	0.000911 ± 0.000559**b**
Flavobacterium	0.000839 ± 0.000322**a**	0.005223 ± 0.003848**a**	0.003922 **a**	0.00245 ± 0.003763**a**	0.000444 ± 0.000641**a**	0.003193 ± 0.002237**a**
Pedobacter	0.008387 ± 0.004902**a**	0.001472 ± 0.001126**b**	0.000199 **b**	0.000157 ± 0.000226**b**	0.001158 ± 0.001441**b**	0.001176 ± 0.000322**b**
Ferruginibacter	0.003522 ± 0.001414**a**	0.002435 ± 0.002128**a**	0.001638 **a**	6.1e-05 ± 8.1e-05**a**	0.000927 ± 0.001606**a**	0.001981 ± 0.000374**a**
Caballeronia	0.00896 ± 0.008306**a**	0.000123 ± 9e-05**a**	8.7e-05 **a**	1.4e-05 ± 2.5e-05**a**	6e-06 ± 1.1e-05**a**	0.000538 ± 0.000479**a**
Nitrosospira	0.000129 ± 4.5e-05**a**	0.000511 ± 0.000397**a**	0.002359 **a**	0.002132 ± 0.001747**a**	0.001321 ± 0.002152**a**	0.002915 ± 0.00065**a**
Aestuariivirga	0.00011 ± 2.4e-05**b**	0.000342 ± 0.000258**b**	0.001933 **ab**	0.001706 ± 0.001141**ab**	0.00024 ± 0.000416**b**	0.004375 ± 0.001944**a**
Burkholderia	0.004547 ± 0.004626**a**	6.2e-05 ± 4.8e-05**a**	0.000545 **a**	0.000286 ± 8.4e-05**a**	1.8e-05 ± 3.1e-05**a**	0.000914 ± 0.0011**a**
Sphingomonas	0.003898 ± 0.003386**a**	0.000102 ± 7.7e-05**a**	0.000171 **a**	4.4e-05 ± 2.1e-05**a**	0.000566 ± 0.000695**a**	0.001084 ± 0.001349**a**

Table S15. The relative abundances of taxa involved in rock weathering at the family level (top 20) in different Damma glacial habitats. Significances were analyzed by one-way analysis of variance (ANOVA) followed by a least significant difference (LSD) test. *P*-values were adjusted using the Benjamini–Hochberg method. Significant differences are annotated by different lowercase letters in bold. Habitats that share the same letter are not significantly different, while those with different letters are significantly different. Values are means ± SD (n=3, except for "Ice" where n=2) followed by lowercase letters indicating significant differences ($P < 0.05$). Snow: surface snow; Stream: proglacial stream water; Ice: subglacial ice; Sub: subglacial sediment; Supra: supraglacial sediment; Soil: recently deglaciated soil.

Family	Habitat					
	**Snow**	**Stream**	**Ice**	**Sub**	**Supra**	**Soil**
Pseudomonadaceae	0.178682 ± 0.151928**a**	0.001937 ± 0.001093**a**	0.001088 **a**	0.004014 ± 0.00629**a**	0.000256 ± 0.000148**a**	0.008881 ± 0.010511**a**
Oxalobacteraceae	0.060897 ± 0.017689**a**	0.011594 ± 0.006774**a**	0.007109 **a**	0.050597 ± 0.038811**a**	0.005003 ± 0.004212**a**	0.012713 ± 0.011716**a**
Comamonadaceae	0.002617 ± 0.00154**b**	0.005943 ± 0.004218**b**	0.005634 **b**	0.101747 ± 0.062873**a**	0.006258 ± 0.003684**b**	0.007206 ± 0.005908**b**
Methylophilaceae	0.000307 ± 7e-05**a**	0.007227 ± 0.00564**a**	0.00057 **a**	0.002731 ± 0.003207**a**	0.050351 ± 0.044558**a**	0.00895 ± 0.001479**a**
Chitinophagaceae	0.007277 ± 0.003126**a**	0.009685 ± 0.007684**a**	0.006556 **a**	0.004489 ± 0.003029**a**	0.024439 ± 0.028928**a**	0.014208 ± 0.00054**a**
Sphingobacteriaceae	0.024854 ± 0.015797**a**	0.005601 ± 0.004126**ab**	0.000746 **b**	0.00025 ± 0.000336**b**	0.002404 ± 0.002074**b**	0.008498 ± 0.00121**ab**
Zoogloeaceae	2.6e-05 ± 2.4e-05**a**	9.3e-05 ± 7.5e-05**a**	0.000468 **a**	0.02767 ± 0.040432**a**	0.002235 ± 0.00364**a**	0.000354 ± 0.000346**a**
Burkholderiaceae	0.021885 ± 0.021086**a**	0.000542 ± 0.000379**a**	0.001435 **a**	0.001394 ± 0.000809**a**	0.000263 ± 0.000261**a**	0.002879 ± 0.003026**a**
Flavobacteriaceae	0.001264 ± 0.000465**a**	0.006232 ± 0.004607**a**	0.003963 **a**	0.00245 ± 0.003763**a**	0.000462 ± 0.000672**a**	0.004828 ± 0.001616**a**
Hymenobacteraceae	0.014578 ± 0.001032**a**	0.000595 ± 0.000257**b**	0.000376 **b**	3.7e-05 ± 1.5e-05**b**	0.000436 ± 0.000374**b**	0.001039 ± 0.000595**b**
Microbacteriaceae	0.01073 ± 0.012396**a**	0.000147 ± 9.4e-05**a**	0.000109 **a**	1.8e-05 ± 1.1e-05**a**	8.1e-05 ± 9.9e-05**a**	0.00088 ± 0.000554**a**
Nitrosomonadaceae	0.000129 ± 4.5e-05**a**	0.000556 ± 0.000436**a**	0.002686 **a**	0.002166 ± 0.001725**a**	0.001397 ± 0.002276**a**	0.003184 ± 0.000609**a**
Sphingomonadaceae	0.004958 ± 0.004066**a**	0.000217 ± 0.000202**a**	0.000293 **a**	0.000799 ± 0.001325**a**	0.000761 ± 0.000632**a**	0.001607 ± 0.001744**a**
Aestuariivirgaceae	0.00011 ± 2.4e-05**b**	0.000342 ± 0.000258**b**	0.001933 **ab**	0.001706 ± 0.001141**ab**	0.00024 ± 0.000416**b**	0.004375 ± 0.001944**a**
Moraxellaceae	5.3e-05 ± 3.5e-05**a**	0.000284 ± 0.000248**a**	0.000111 **a**	0.003047 ± 0.002397**a**	1.3e-05 ± 1.5e-05**a**	0.003997 ± 0.002992**a**
Spirosomaceae	0.00031 ± 0.000158**a**	0.003121 ± 0.00275**a**	0.000136 **a**	2.7e-05 ± 3.6e-05**a**	0.001824 ± 0.003054**a**	0.000827 ± 0.000332**a**
Gallionellaceae	0.000112 ± 1e-04**a**	0.002054 ± 0.00131**a**	8e-04 **a**	0.000331 ± 0.000116**a**	0.00148 ± 0.001653**a**	0.00115 ± 0.000201**a**
Perlucidibacaceae	3.4e-05 ± 4.6e-05**a**	0.000235 ± 0.000244**a**	6.4e-05 **a**	0.00345 ± 0.003971**a**	5.3e-05 ± 8.7e-05**a**	0.001607 ± 0.001177**a**
Cytophagaceae	0.000304 ± 0.000322**a**	0.000244 ± 0.000205**a**	0.000217 **a**	1.3e-05 ± 1.1e-05**a**	0.003992 ± 0.006847**a**	0.000433 ± 3.1e-05**a**
Sphaerotilaceae	0.000131 ± 7.6e-05**a**	7.2e-05 ± 5.5e-05**a**	0.000775 **a**	0.001728 ± 0.00269**a**	0.000581 ± 0.000879**a**	0.000886 ± 0.000882**a**

Table S16. Summary of the functional genes annotated using eggNOG database that were significantly overrepresented/underrepresented in one specific habitat. Only significantly overrepresented/underrepresented functional genes annotated using eggNOG database in one specific habitat compared with other habitats in the Damma glacier environment are listed. The table is ordered by gene categories followed by the corresponding individual genes within the stated gene function). Genes at the individual/family level are formatted as gene ID followed by their annotated functions. "↑" means the genes were significantly more abundant (log2-fold change (LFC>0, P<0.05) in the specific habitat relative to in other habitats, "↓" means the genes were significantly less abundant in the specific habitat relative to in other habitats (LFC<0, P<0.05). For example, "K" is overrepresented in Snow, and COG1675 within Category "K", which has the genetic potential to encode TFIIE alpha subunit, is overrepresented in Snow habitat. * K: Transcription, N: Cell motility, G: Carbohydrate transport and metabolism, C: Energy production and conversion, F: Nucleotide transport and metabolism, L: Replication, recombination and repair, O: Posttranslational modification, protein turnover, chaperones, T: Signal transduction mechanisms, U: Intracellular trafficking, secretion, and vesicular transport, J: Translation, ribosomal structure biogenesis. Snow: surface snow; Stream: proglacial stream water; Ice: subglacial ice; Sub: subglacial sediment; Supra: supraglacial sediment; Soil: recently deglaciated soil.

Habitat	Category*	Gene ID	Function
**Snow**	K ↑	COG1675	TFIIE alpha subunit
		COG3160	Anti-RNA polymerase sigma 70 factor
		COG3283	transcriptional regulator TyrR;Transcriptional regulator
		COG4650	Transcriptional regulator
		COG4957	Transcriptional regulator
		COG5157	RNA pol II accessory factor, Cdc73 family
	N ↑	COG1261	Flagella basal body P-ring formation protein
		COG1334	Flagellar protein;flagellar protein FlaG
		COG1580	Flagellar basal body-associated protein flil
		COG1843	Flagellar hook capping protein;flagellar basal body rod modification protein
		COG2063	Flagellar L-ring protein
		COG4787	Flagellar basal-body rod protein FlgF
	G ↑	COG2213	PTS system lactose cellobiose-specific transporter subunit IIB
		COG2814	Major facilitator superfamily MFS_1
		COG3734	K00883 2-dehydro-3-deoxygalactonokinase EC 2.7.1.58
		COG4158	Monosaccharide-transporting ATPase
		COG4580	Involved in the transport of maltose and maltodextrins (By similarity)
		COG4668	Phosphoenolpyruvate-dependent sugar phosphotransferase system, EIIA 2
	C ↓	COG0374	Nickel-dependent hydrogenase large subunit
		COG1013	2-oxoglutarate ferredoxin oxidoreductase subunit beta
		COG1014	Indolepyruvate oxidoreductase subunit B
		COG1229	Formylmethanofuran dehydrogenase, subunit A
		COG1625	Oxidoreductase
		COG3260	NADH ubiquinone oxidoreductase 20 kDa subunit
		COG3261	NADH-ubiquinone oxidoreductase chain 49kDa
		COG3383	Formate dehydrogenase alpha subunit
		COG3894	Iron-sulfur cluster-binding protein
	F ↓	COG0813	Purine nucleoside phosphorylase DeoD-type
	L ↓	COG1943	Transposase IS200-family protein

		COG3039	Transposase
	G ↓	COG3298	3'-5' exonuclease
		COG3464	Transposase, TnpA from firmNOG
		COG5421	Inherit from bactNOG: Transposase-like protein
Stream	F ↑	COG0125	Deoxythymidylate kinase (thymidylate kinase)
		COG0207	Biosynthesis of dTMP
Ice	F ↑	COG0127	Pyrophosphatase
		COG0213	Pyrimidine-nucleoside phosphorylase
	O ↑	COG1030	NfeD-like C-terminal, partner-binding;Inherit from bactNOG
		COG2192	K00612 carbamoyltransferase EC 2.1.3
		COG3187	META domain protein
		COG1102	Cytidine monophosphate kinase
	K ↓	COG1759	IMP biosynthesis enzyme PurP domain protein
		COG4977	Transcriptional regulator, ARAC family
	N ↓	COG5555	Metallophosphoesterase
	T ↓	COG2200	Response regulator receiver modulated diguanylate phosphodiesterase
		COG3290	Sensory histidine kinase DcuS
		COG3448	HPP family
		COG4565	Transcriptional regulatory protein
		COG5212	Cyclic-AMP phosphodiesterase
Sub	U ↑	COG3167	Assembly protein PilO
		COG4968	Type 4 fimbrial biogenesis transmembrane protein
	N ↑	COG1345	Flagellar hook-associated
		COG1580	Flagellar basal body-associated protein flil
		COG4787	Flagellar basal-body rod protein FlgF
	T ↑	COG3073	Anti sigma-E protein RseA
		COG3434	Signal transduction protein
		COG5001	Diguanylate cyclase phosphodiesterase
	F ↓	COG1351	FAD-dependent thymidylate synthase
		COG1435	Thymidine kinase
		COG1437	Adenylate cyclase
		COG4360	ATP adenylyltransferase
	G ↓	COG0362	Catalyzes the oxidative decarboxylation
**Supra**	G ↓	COG4130	Xylose isomerase-like TIM barrel
		COG4409	Inherit from bctoNOG: Glycosyl hydrolase BNR repeat-containing protein
	U ↓	COG3736	Type IV secretion system protein
		COG3149	General secretion pathway M protein
		COG5043	Vacuolar protein
		COG5158	Syntaxin binding protein
		COG5307	Guanine Sec7 domain protein
**Soil**	J ↑	COG1206	Catalyzes the folate-dependent formation of 5-methyl- uridine at position 54 (M-5-U54) in all tRNAs (By similarity)
		COG2302	S4 domain protein
	N ↓	COG1345	Flagellar hook-associated protein 2 N-terminus
		COG1419	Flagellar biosynthesis regulator FlhF
		COG1580	Flagellar basal body-associated protein flil
	K ↓	COG2944	Transcriptional Regulator, XRE family

Table S17. Taxonomy and relative abundance (%) associated to N-fixing genes in the studied Damma glacial habitats. Snow: surface snow; Stream: proglacial stream water; Ice: subglacial ice; Sub: subglacial sediment; Supra: supraglacial sediment; Soil: recently deglaciated soil.

Contig	Phylum	Snow	Stream	Ice	Sub	Supra	Soil
k141_809813	Cyanobacteriota	0.00E+00	0.00E+00	0.00E+00	0.00E+00	0.00E+00	2.22E-04
k141_495722	Cyanobacteriota	3.26E-05	1.37E-05	0.00E+00	0.00E+00	8.81E-06	5.75E-04
k141_2528172	Cyanobacteriota	3.63E-06	1.37E-05	0.00E+00	0.00E+00	0.00E+00	2.42E-04
k141_1262873	Cyanobacteriota	2.18E-05	1.71E-05	8.74E-06	0.00E+00	5.87E-06	6.85E-04
k141_2207792	Cyanobacteriota	0.00E+00	0.00E+00	0.00E+00	0.00E+00	0.00E+00	3.63E-04
k141_785123	Cyanobacteriota	0.00E+00	1.71E-05	0.00E+00	0.00E+00	0.00E+00	3.53E-04
k141_1553397	Cyanobacteriota	0.00E+00	0.00E+00	0.00E+00	0.00E+00	0.00E+00	1.92E-04
k141_797117	Cyanobacteriota	1.09E-05	0.00E+00	0.00E+00	0.00E+00	0.00E+00	1.71E-04
k141_2195665	Cyanobacteriota	0.00E+00	1.71E-05	0.00E+00	0.00E+00	0.00E+00	1.11E-04
k141_1784507	Bacillota	7.25E-06	0.00E+00	3.50E-05	1.20E-04	8.81E-06	0.00E+00
k141_2477291	Bacillota	0.00E+00	1.03E-05	8.74E-06	9.66E-05	0.00E+00	0.00E+00
k141_2461728	Bacillota	0.00E+00	6.84E-06	8.74E-06	1.89E-05	2.34E-03	0.00E+00
k141_1519328	Pseudomonadota	0.00E+00	0.00E+00	5.68E-05	1.46E-04	0.00E+00	0.00E+00
k141_2321526	Pseudomonadota	0.00E+00	5.47E-05	0.00E+00	0.00E+00	9.40E-05	2.22E-04
k141_1593996	Candidatus Shapirobacteria	0.00E+00	0.00E+00	4.37E-06	0.00E+00	0.00E+00	2.72E-04
k141_1441328	Euryarchaeota	0.00E+00	0.00E+00	5.24E-05	0.00E+00	5.87E-06	0.00E+00
k141_130054	Thermodesulfobacteriota	0.00E+00	0.00E+00	8.74E-06	0.00E+00	1.76E-05	0.00E+00
k141_85295	Unclassified	7.25E-06	0.00E+00	4.37E-06	5.89E-05	0.00E+00	0.00E+00
k141_1686040	Unclassified	0.00E+00	0.00E+00	0.00E+00	1.18E-05	0.00E+00	1.01E-05
k141_1552849	Unclassified	0.00E+00	0.00E+00	5.24E-05	5.75E-04	0.00E+00	4.03E-05
k141_2111180	Unclassified	0.00E+00	0.00E+00	3.06E-05	0.00E+00	3.23E-05	0.00E+00
k141_932272	Unclassified	0.00E+00	6.84E-06	4.37E-06	0.00E+00	2.94E-06	6.05E-05

Supplementary Figures

Microbial genetic potential differs among cryospheric habitats of the Damma glacier

Maomao Feng, Serina Robinson, Weihong Qi, Arwyn Edwards, Beat Stierli, Marcel Van der Heijden, Beat Frey, Gilda Varliero

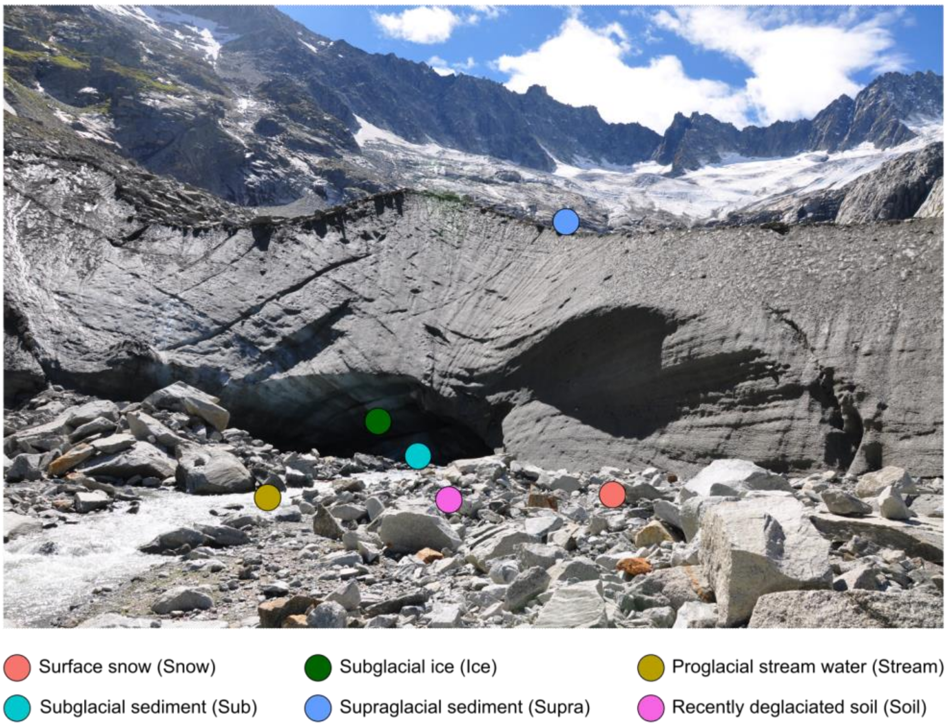

Figure S1. Picture of the forefront of the Damma glacier, highlighting the six habitats sampled for this study.

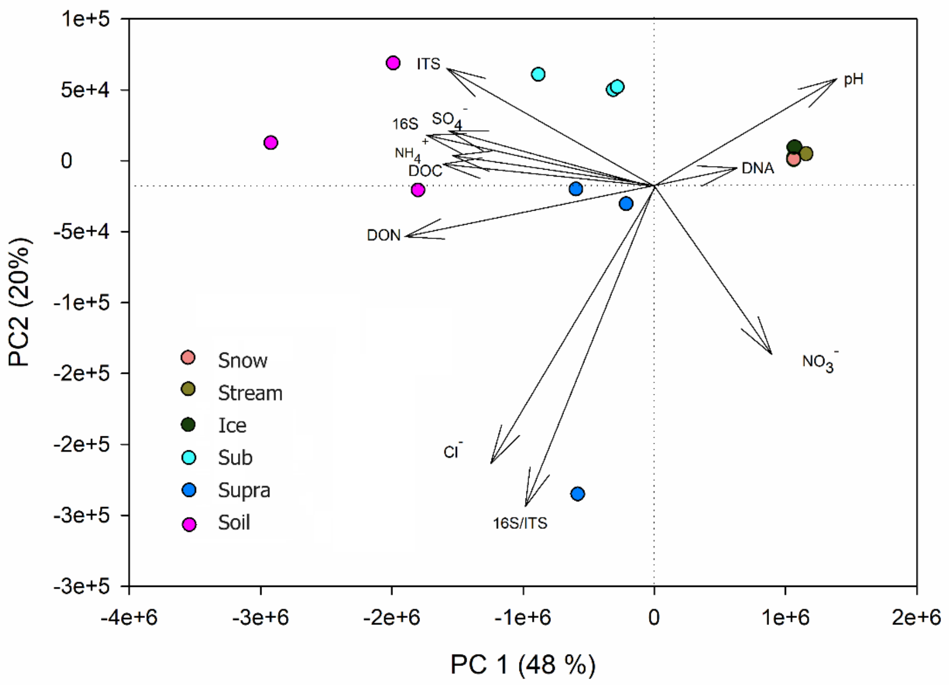

Figure S2. PCA ordination of environmental variables for samples from different habitats

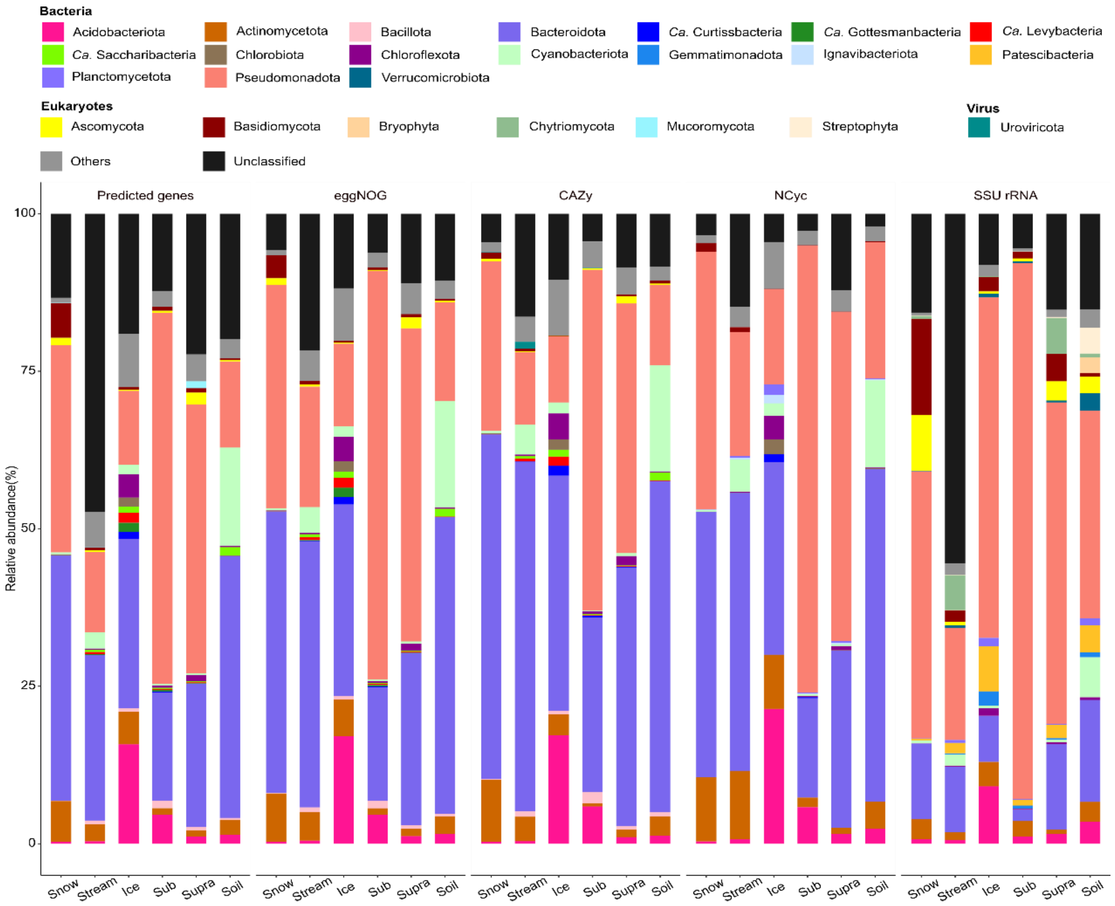

Figure S3. Taxonomic composition of the microbiomes in six Damma glacial habitats based on predicted, functional (annotated using eggNOG, CAZy and NCyc databases), and ribosomal genes (SSU). Relative abundance is the mean of three replicates (only two replicates for Ice samples). Only phyla with a relative abundance >1% in at least one habitat are shown. Snow: surface snow; Stream: proglacial stream water; Ice: subglacial ice; Sub: subglacial sediment; Supra: supraglacial sediment; Soil: recently deglaciated soil.

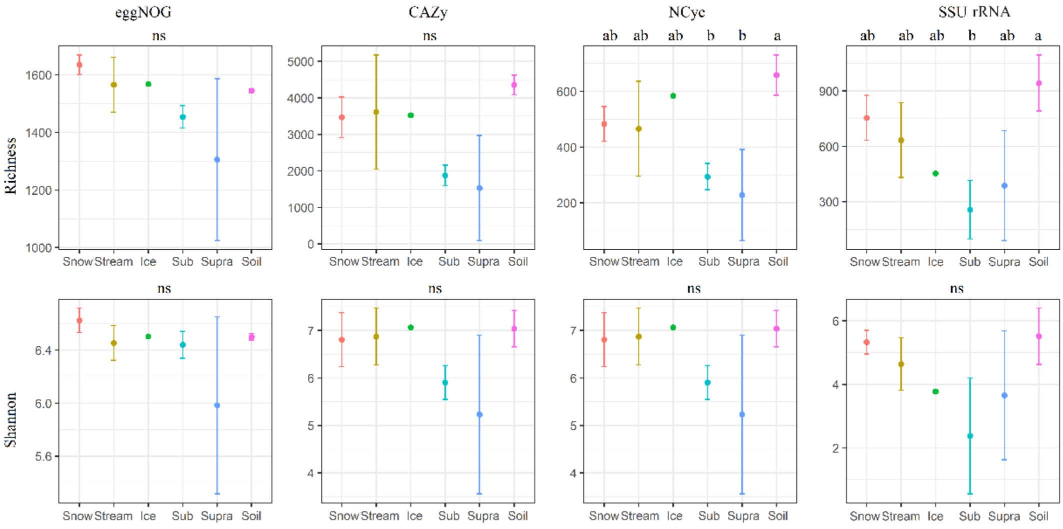

Figure S4. Changes in the alpha-diversity of functional (annotated using eggNOG, CAZy and NCyc databases) and ribosomal (SSU) genes among six glacial habitats. Dots indicate the mean and the lines indicate the standard deviation (*n*=3, except for "Ice" where *n*=2). Significance was calculated using one-way analysis of variance (ANOVA) followed by a least significant difference (LSD) test. *P*-values were corrected for multiple testing with the Benjamini–Hochberg method. Significant differences between habitats are annotated by different lowercase letters. Snow: surface snow; Stream: proglacial stream water; Ice: subglacial ice; Sub: subglacial sediment; Supra: supraglacial sediment; Soil: recently deglaciated soil.

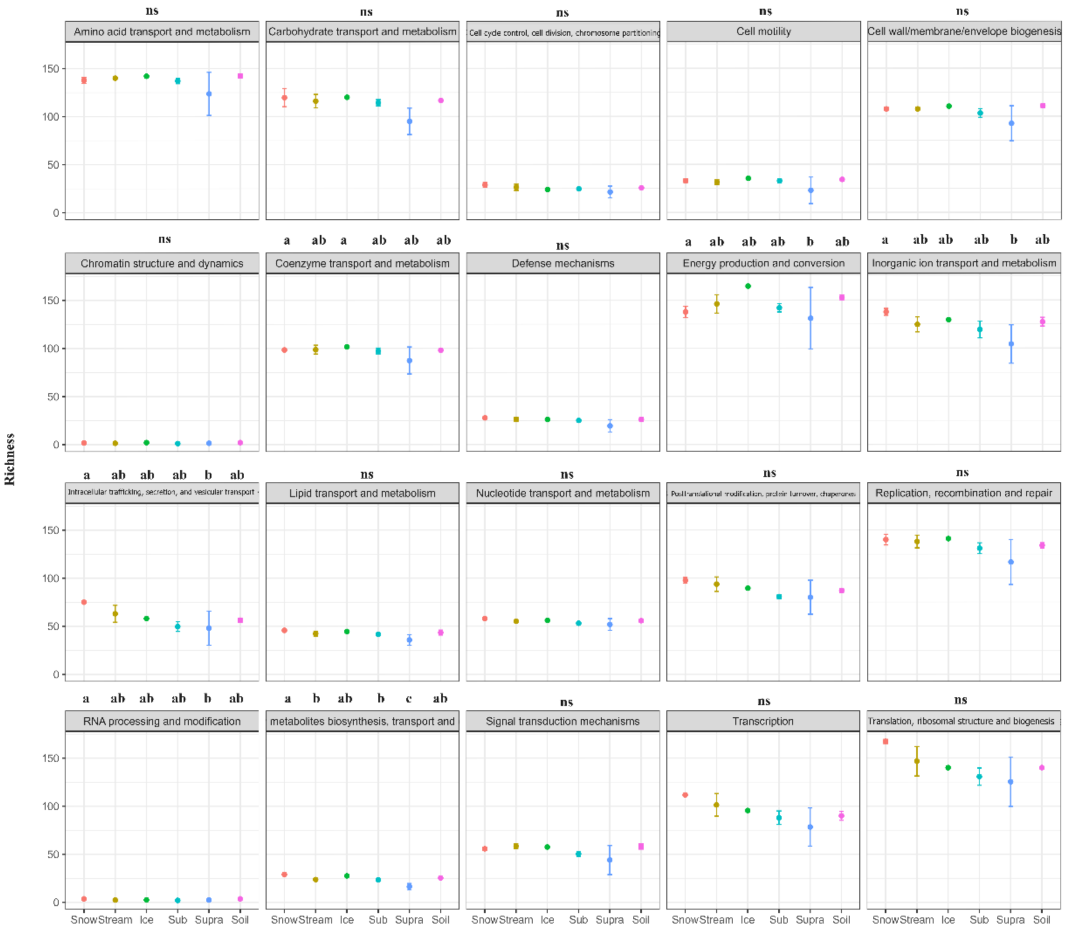

Figure S5. Alpha-diversity (richness) of functional categories annotated using the eggNOG database in different Damma glacial habitats. Dots indicate the mean, and the lines indicate the standard deviation (n=3, except for "Ice" where n=2). Significance was calculated using one-way analysis of variance (ANOVA) followed by a least significant difference (LSD) test. P-values were corrected for multiple testing with the Benjamini–Hochberg method. Significant differences between habitats are annotated by different lowercase letters (ns = not significant). Snow = surface snow; Stream = proglacial stream water; Ice = subglacial ice; Sub = subglacial sediment; Supra = supraglacial sediment; Soil = recently deglaciated soil.

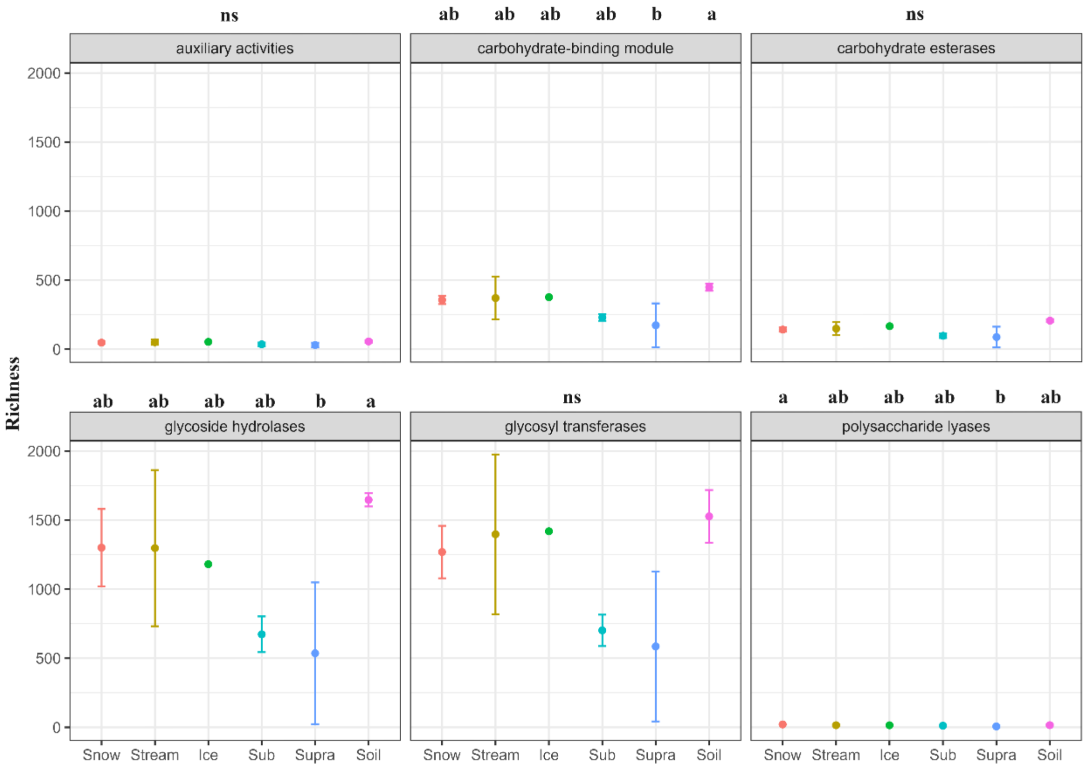

Figure S6. Alpha-diversity (richness) of functional categories annotated using the CAZy database in different Damma glacial habitats. Dots indicate the mean, and the lines indicate the standard deviation (*n*=3, except for "Ice" where *n*=2). Significance was calculated using one-way analysis of variance (ANOVA) followed by a least significant difference (LSD) test. *P*-values were corrected for multiple testing with the Benjamini–Hochberg method. Significant differences between habitats are annotated by different lowercase letters (ns = not significant). Snow: surface snow; Stream: proglacial stream water; Ice: subglacial ice; Sub: subglacial sediment; Supra: supraglacial sediment; Soil: recently deglaciated soil.

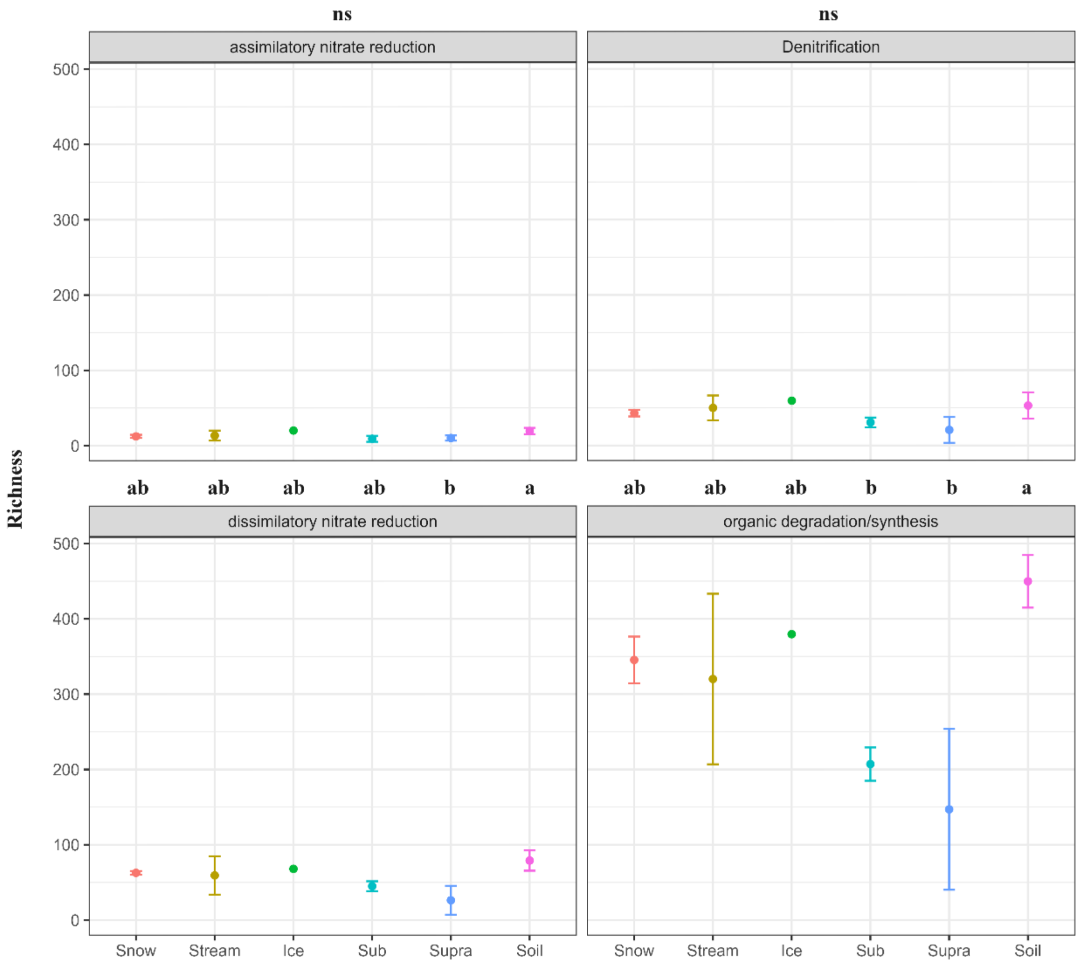

Figure S7. Alpha-diversity (richness) of functional categories annotated using the NCyc database in different Damma glacial habitats. Dots indicate the mean, and the lines indicate the standard deviation (*n*=3, except for "Ice" where *n*=2). Significance was calculated using one-way analysis of variance (ANOVA) followed by a least significant difference (LSD) test. *P*-values were corrected for multiple testing with the Benjamini–Hochberg method. Significant differences between habitats are annotated by different lowercase letters (ns = not significant). Snow: surface snow; Stream: proglacial stream water; Ice: subglacial ice; Sub: subglacial sediment; Supra: supraglacial sediment; Soil: recently deglaciated soil.

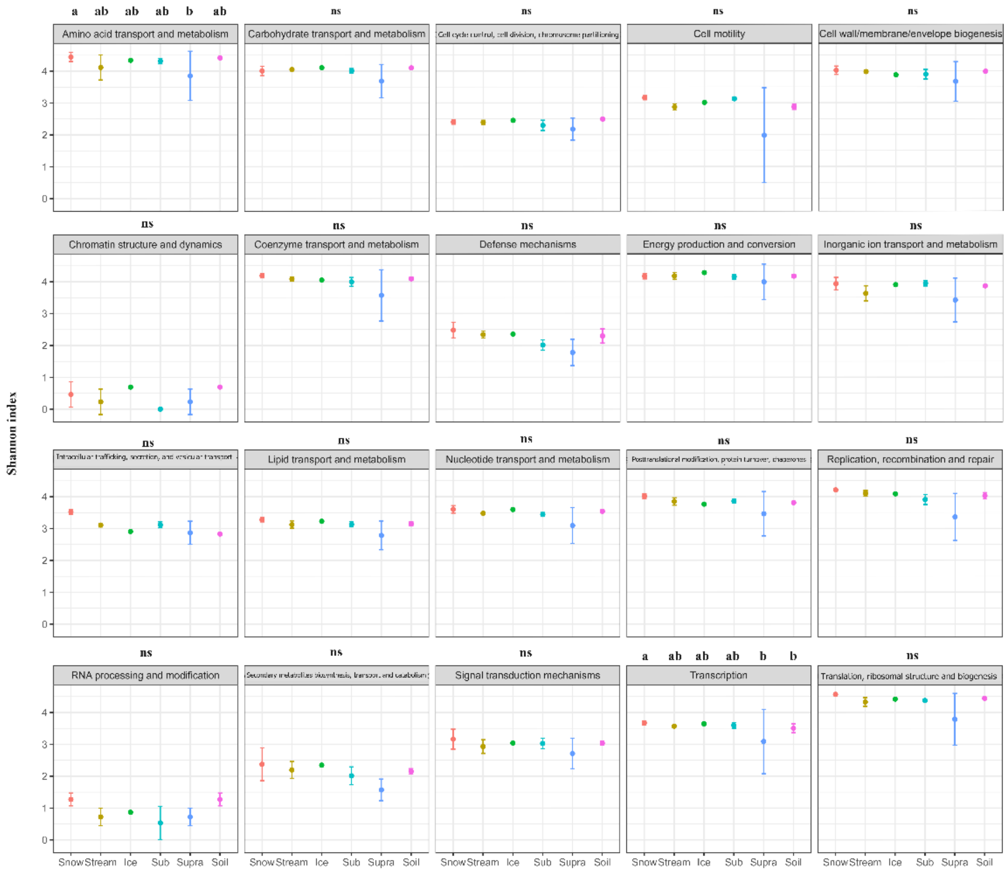

Figure S8. Alpha-diversity (Shannon index) of functional categories annotated using the eggNOG database in different Damma glacial habitats. The specific functional description of the categories is the same as in Figure S5. Dots indicate the mean, and the lines indicate the standard deviation (*n*=3, except for “Ice” where *n*=2). Significance was calculated using one-way analysis of variance (ANOVA) followed by a least significant difference (LSD) test. *P*-values were corrected for multiple testing with the Benjamini–Hochberg method. Significant differences between habitats are annotated by different lowercase letters (ns = not significant). Snow = surface snow; Stream = proglacial stream water; Ice = subglacial ice; Sub = subglacial sediment; Supra = supraglacial sediment; Soil = recently deglaciated soil

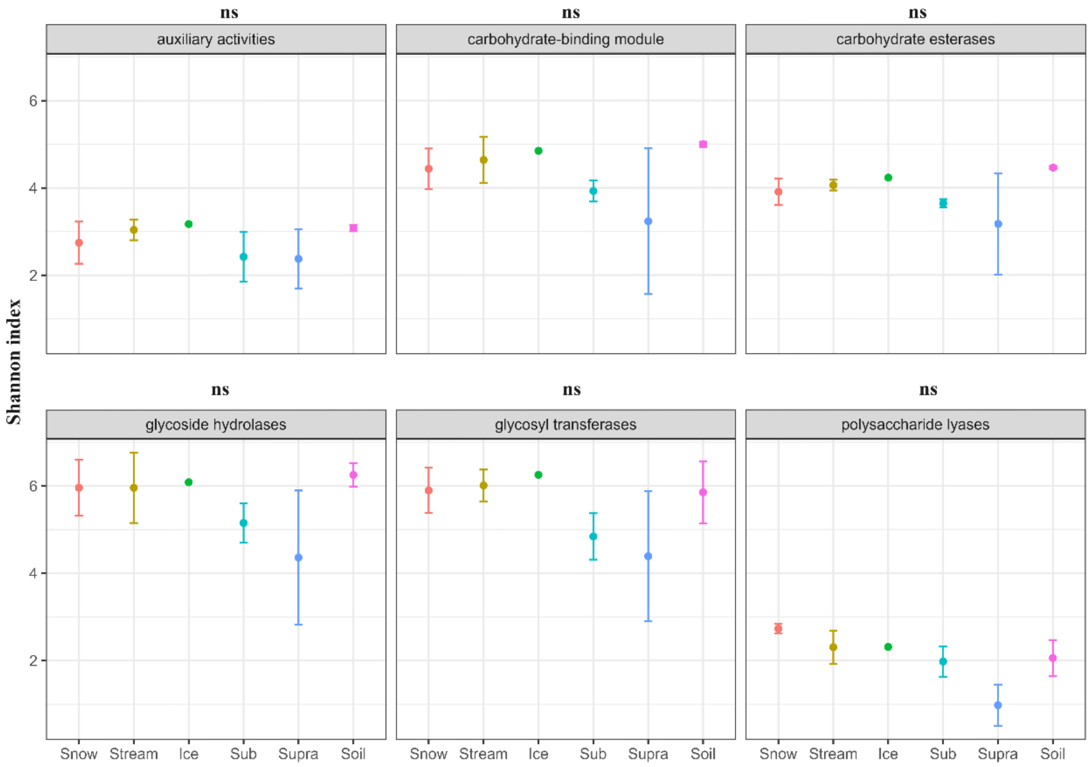

Figure S9. Alpha-diversity (Shannon index) of functional categories annotated using the CAZy database in different Damma glacial habitats. Dots indicate the mean, and the lines indicate the standard deviation (n=3, except for "Ice" where n=2). Significance was calculated using one-way analysis of variance (ANOVA) followed by a least significant difference (LSD) test. P-values were corrected for multiple testing with the Benjamini–Hochberg method. Significant differences between habitats are annotated by different lowercase letters (ns = not significant). Snow: surface snow; Stream: proglacial stream water; Ice: subglacial ice; Sub: subglacial sediment; Supra: supraglacial sediment; Soil: recently deglaciated soil.

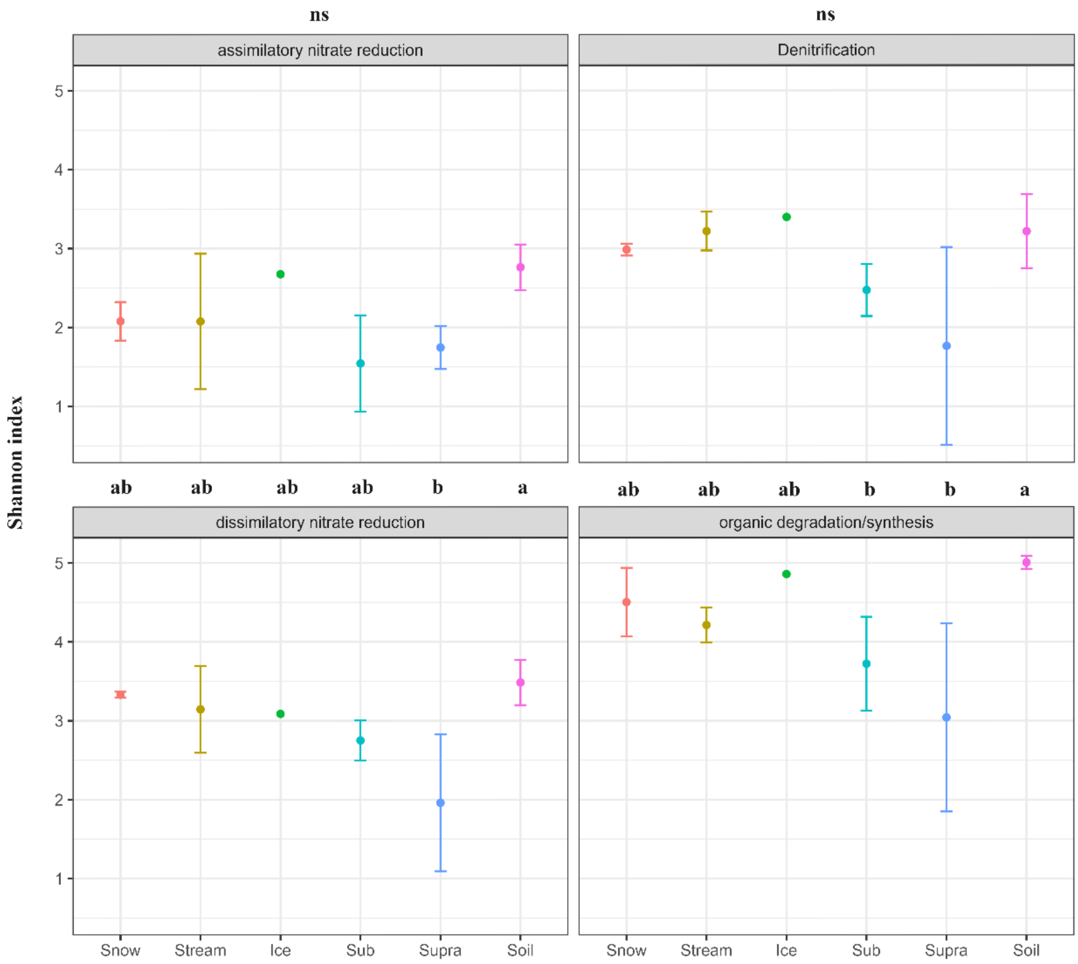

Figure S10. Alpha-diversity (Shannon index) of functional categories annotated using the NCyc database in different Damma glacial habitats. Dots indicate the mean, and the lines indicate the standard deviation (*n*=3, except for "Ice" where *n*=2). Significance was calculated using one-way analysis of variance (ANOVA) followed by a least significant difference (LSD) test. *P*-values were corrected for multiple testing with the Benjamini–Hochberg method. Significant differences between habitats are annotated by different lowercase letters (ns = not significant). ANR: assimilatory nitrate reduction; DNR: dissimilatory nitrate reduction; OD&S: organic degradation/synthesis; Snow: surface snow; Stream: proglacial stream water; Ice: subglacial ice; Sub: subglacial sediment; Supra: supraglacial sediment; Soil: recently deglaciated soil.

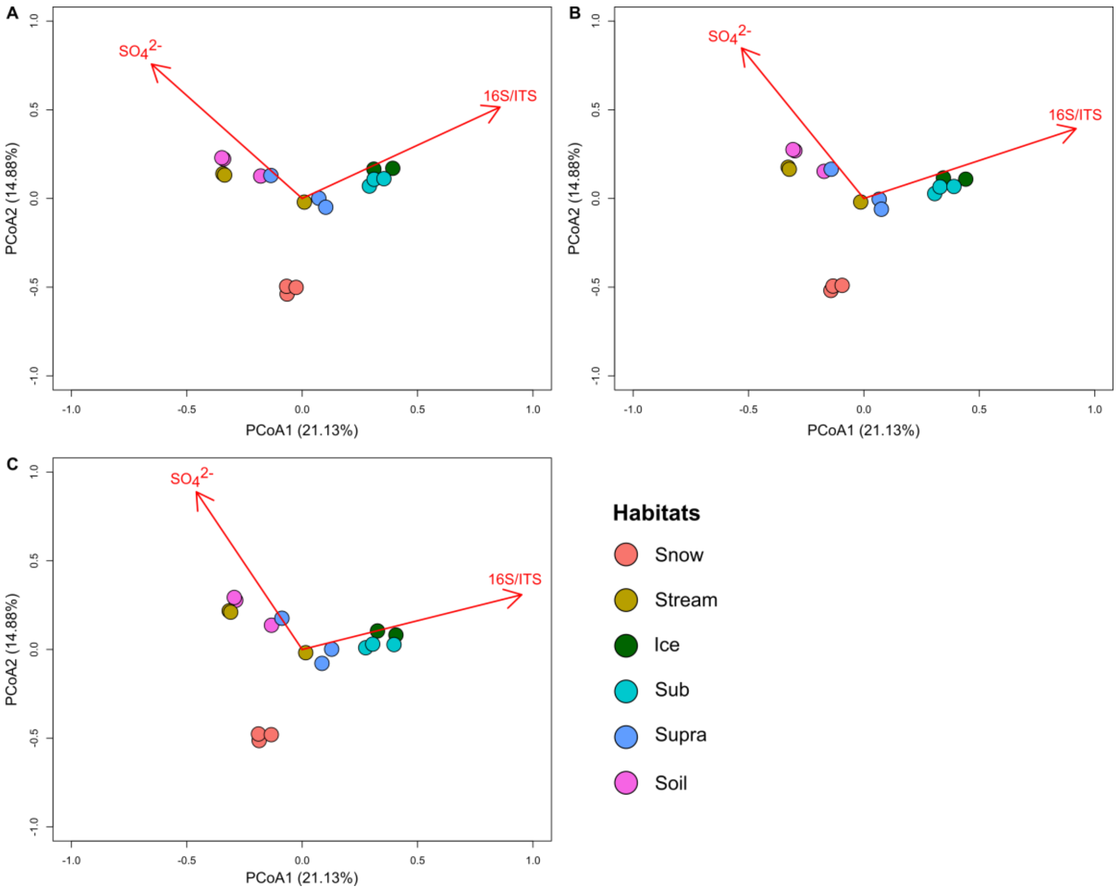

Figure S11. Changes in the structure of functional genes annotated with eggNOG (A), CAZy (B) and NCyc (C) across six Damma glacial habitats. The percentage of the variation explained by each principal coordinates analysis (PCoA) axis is given in brackets. Vectors represent a regression of soil physico-chemical and biotic parameters against the PCoA ordination scores. All the vectors displayed here had significant correlations (*P*<0.05) with microbial functional and ribosomal gene structures Snow: surface snow; Stream: proglacial stream water; Ice: subglacial ice; Sub: subglacial sediment; Supra: supraglacial sediment; Soil: barren soil. SO_4^{2-}: sulfate; Cl^-: chloride; 16S/ITS: ratio of copy numbers of bacterial 16S rRNA and fungal ITS.

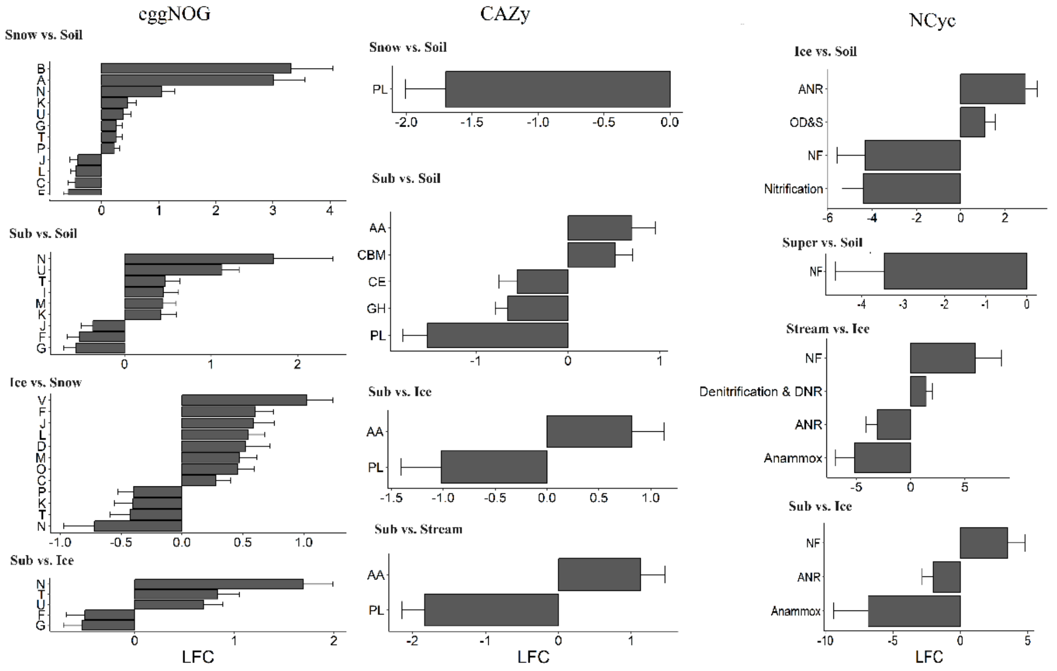

Figure S12. Differentially abundant functional genes between Damma glacier habitats at functional category/class/family level. Bars represent log2-fold changes (LFCs) in functional genes annotated with eggNOG, CAZy and NCyc database, aggregated over functional categories that differed in abundance (P< 0.01) between two habitats. Positive LFCs mean that genes are enriched in the former soils, e.g., in (a) functional category "B" were more abundant in Snow than Soil habitat.

[eggnog]

Information storage and processing:

A: RNA processing and modification; B: chromatin structure and dynamics; K: transcription; L: replication, recombination and repair; J: translation, ribosomal structure and biogenesis

Cellular processes and signaling:

D: cell cycle control, cell division and chromosome partitioning: M: cell wall/membrane/envelope biogenesis; N: cell motility; O: posttranslational modification, protein turnover and chaperones; T: signal transduction mechanisms; U: intracellular trafficking, secretion and vesicular transport; V: defense mechanisms

Metabolism:

C: energy production and conversion; E: amino acid transport and metabolism; F: nucleotide transport and metabolism; G: carbohydrate transport and metabolism; H: coenzyme transport and metabolism; I: lipid transport and metabolism; P: inorganic ion transport and metabolism; Q: secondary metabolite biosynthesis, transport and catabolism

[CAZy] AA: auxiliary activities; CBM: carbohydrate-binding module; GH：glycoside hydrolases; GT: glycosyl transferase; PL: polysaccharide lyase

[NCyc] ANR: assimilatory nitrate reduction; DNR: dissimilatory nitrate reduction; NF: nitrogen fixation; OD&S: organic degradation/synthesis

Snow: surface snow; Stream: proglacial stream water; Ice: subglacial ice; Sub: subglacial sediment; Supra: supraglacial sediment; Soil: barren soil

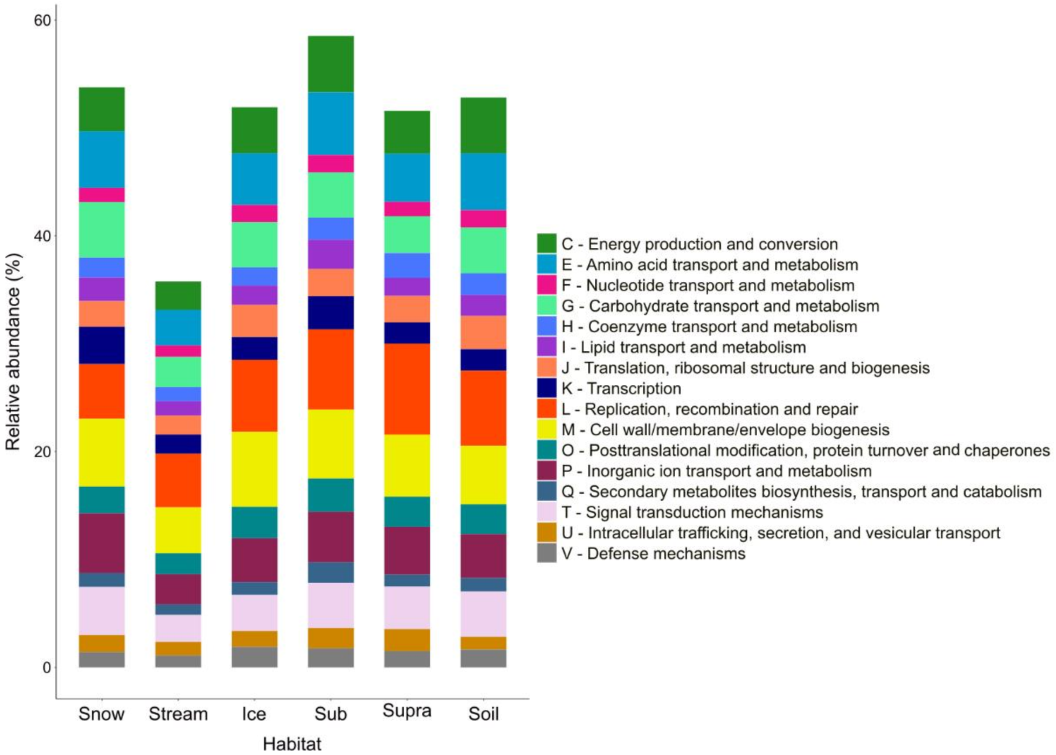

Figure S13. Relative abundance of eggNOG categories in six habitats (only COGs are included) in different Damma glacial habitats. Only categories representing at least 1% in at least one habitat are displayed. Category S ("Function unknown") is not displayed. Bars represent the mean of three replicates (except for "Ice", where only two samples were used). Snow: surface snow; Ice: subglacial ice; Sub: subglacial sediment; Stream: proglacial stream water; Supra: supraglacial sediment; Soil: recently deglaciated soil.

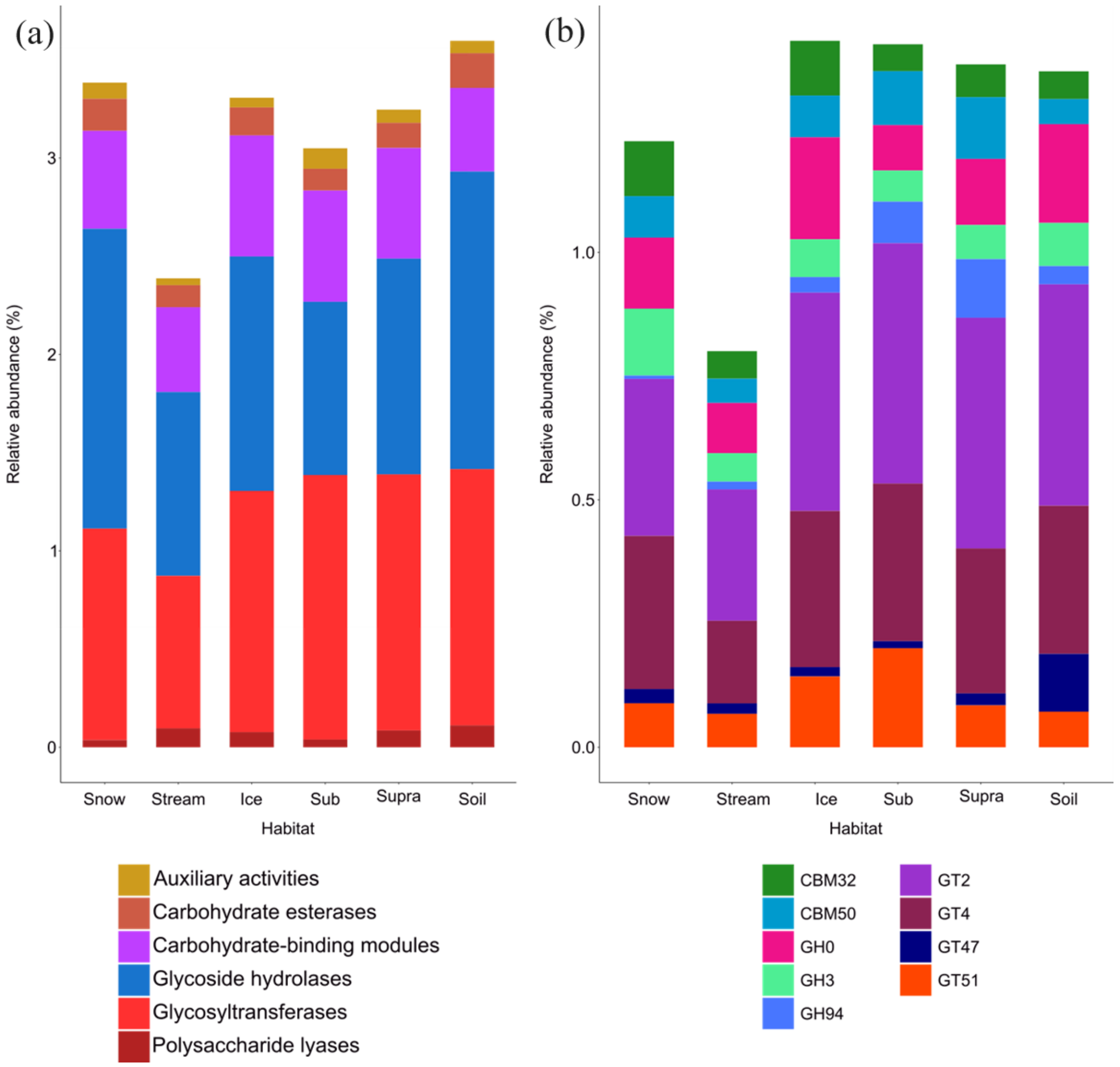

Figure S14. Relative abundance of C-cycling genes at the class (a) and the family (b) level in different Damma glacial habitats. Bars represent the mean of three replicates (except for the "Ice", where only two samples were used). Only families with an abundance >0.1% in at least one habitat are displayed. Snow: surface snow; Stream: proglacial stream water; Ice: subglacial ice; Sub: subglacial sediment; Supra: supraglacial sediment; Soil: recently deglaciated soil.

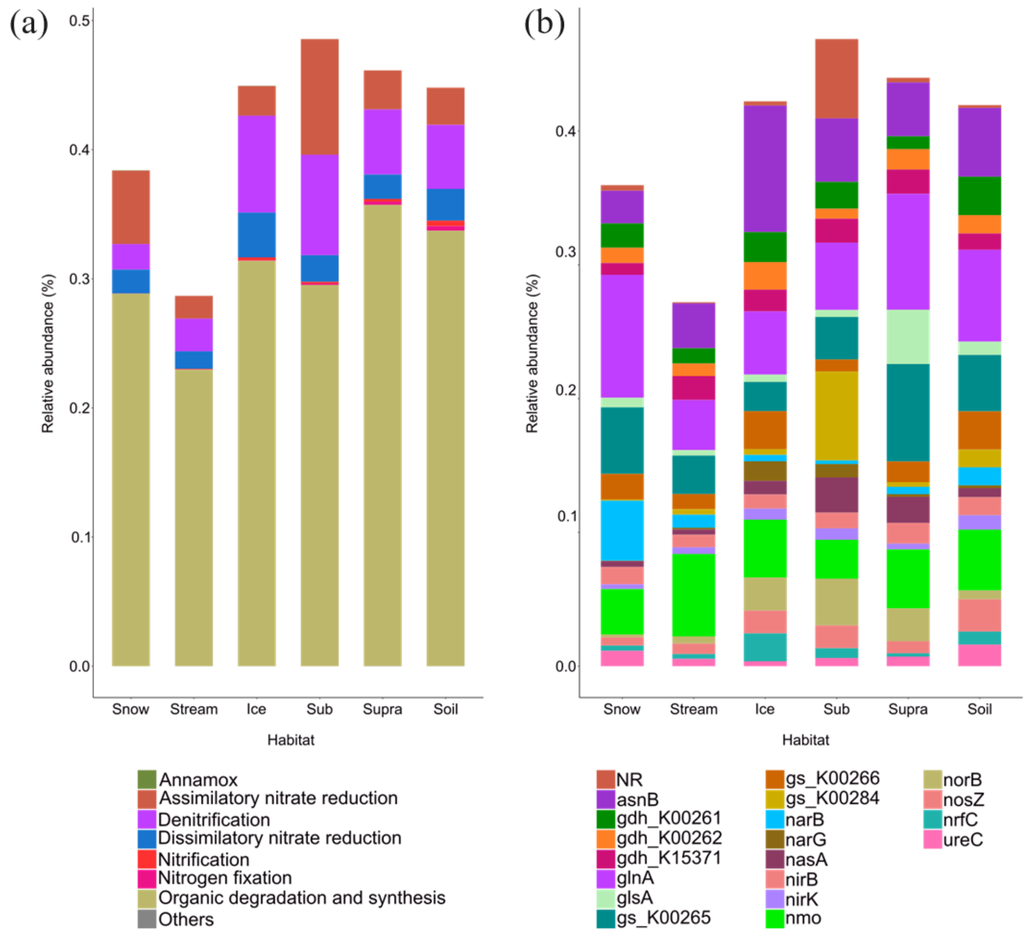

Figure S15. Relative abundance of N-cycling genes at the family (a) and gene (b) level in different Damma glacial habitats. Bars represent the mean of three replicates (except for "Ice, where only two samples were used). Only families with an abundance >0.1% in at least one habitat are displayed. Snow: surface snow; Stream: proglacial stream water; Ice: subglacial ice; Sub: subglacial sediment; Supra: supraglacial sediment; Soil: recently deglaciated soil.
